# Wild blueberry proanthocyanidins shape distinct gut microbiota profile and influence glucose homeostasis and intestinal phenotypes in high-fat high-sucrose fed mice

**DOI:** 10.1038/s41598-020-58863-1

**Published:** 2020-02-10

**Authors:** Maria-Carolina Rodríguez-Daza, Laurence Daoust, Lemia Boutkrabt, Geneviève Pilon, Thibault Varin, Stéphanie Dudonné, Émile Levy, André Marette, Denis Roy, Yves Desjardins

**Affiliations:** 1 Institute of Nutrition and Functional Foods (INAF), Laval University, Québec, QC Canada; 2 Food Science Department, Faculty of Agriculture and Food, Laval University, Québec, QC Canada; 3 Department of Medicine, Faculty of Medicine, Cardiology Axis of the Quebec Heart and Lung Institute, Québec, QC Canada

**Keywords:** Next-generation sequencing, Colon, Microbiota, Dysbiosis, Obesity

## Abstract

Blueberries are a rich source of polyphenols, widely studied for the prevention or attenuation of metabolic diseases. However, the health contribution and mechanisms of action of polyphenols depend on their type and structure. Here, we evaluated the effects of a wild blueberry polyphenolic extract (WBE) (*Vaccinium angustifolium* Aiton) on cardiometabolic parameters, gut microbiota composition and gut epithelium histology of high-fat high-sucrose (HFHS) diet-induced obese mice and determined which constitutive polyphenolic fractions (BPF) was responsible for the observed effects. To do so, the whole extract was separated in three fractions, F1) Anthocyanins and phenolic acids, F2) oligomeric proanthocyanidins (PACs), phenolic acids and flavonols (PACs degree of polymerization DP < 4), and F3) PACs polymers (PACs DP > 4) and supplied at their respective concentration in the whole extract. After 8 weeks, WBE reduced OGTT AUC by 18.3% compared to the HFHS treated rodents and the F3 fraction  contributed the most to this effect. The anthocyanin rich F1 fraction did not reproduce this response. WBE and the BPF restored the colonic mucus layer. Particularly, the polymeric PACs-rich F3 fraction increased the mucin-secreting goblet cells number. WBE caused a significant 2-fold higher proportion of *Adlercreutzia equolifaciens* whereas oligomeric PACs-rich F2 fraction increased by 2.5-fold the proportion of *Akkermansia muciniphila*. This study reveals the key role of WBE PACs in modulating the gut microbiota and restoring colonic epithelial mucus layer, providing a suitable ecological niche for mucosa-associated symbiotic bacteria, which may be crucial in triggering health effects of blueberry polyphenols.

## Introduction

Wild blueberries *(Vaccinium angustifolium* Aiton) are recognized as a rich source of bioactive phenolic compounds, the consumption of which has been associated with the attenuation of metabolic disorders through its beneficial action on glucose homeostasis and the reduction the oxidative stress and intestinal inflammation^1,2^. Until now, these positive effects have predominantly been attributed to flavonoids and in particular to anthocyanins^3,4^. However, wild blueberry has a complex profile of bioactive polyphenols; in addition to anthocyanins, it contains chlorogenic acid, and oligomers and polymers of flavan-3-ols, such as proanthocyanidins (PACs)^5^. Interestingly, there are increased evidences showing that PACs can attenuate the progression of metabolic syndrome, including type 2 diabetes and obesity^6^. Pre-clinical studies have suggested different mechanisms by which PACs regulate metabolic health, ranging from immunomodulatory signaling, stimulation of glucose and lipid metabolism, dampening of metabolic endotoxemia, as well as through inhibition of digestives enzymes^7–9^. However, since high molecular weight PACs are poorly absorbed in the small intestine^10^, they reach the large intestine where they interact with the gut microbiota and exert prebiotic-like effects^11^.

A disrupted intestinal homeostasis and alterations in the gut microbiota diversity and taxonomic composition (i.e., dysbiosis) have been found to affect intestinal immune response^12^ and contribute to the pathogenesis of obesity and type 2 diabetes; it may therefore represent novel risk factors for these metabolic disorders. Likewise, gut epithelial inflammation and altered mucus layer lead to increased epithelial permeability and are specifically associated with an altered gut microbiota^13–15^. Among the life style factors affecting gut microbial diversity, diet quality is of paramount importance and contribute significantly to the determination of gut microbiota composition^16^. In this context, berry polyphenols, including wild blueberry, have recently been shown to significantly promote the abundance of beneficial bacteria involved in the attenuation of obesity-associated disorders and dysbiosis^17,18^. Indeed, in rats fed a high-fat diet, the supplementation of blueberry powder for 8-weeks improved insulin sensitivity and systemic inflammation in association with major changes in gut microbiota composition^19^. In human, a cross-over dietary intervention study revealed prebiotic-like effects of wild blueberry on the *Bifidobacterium* spp. relative abundance^20^. Furthermore, a highbush blueberry polyphenolic fraction and an anthocyanin-enriched fraction were found to attenuate gut barrier dysfunction induced by *E*. *coli* in a model of Caco-2 epithelial cell culture^21^. Whilst those findings have been obtained using different methodologies and model systems, together they provide strong evidences to suggest that wild blueberry polyphenols can selectively modulate the gut microbiota composition and prevent intestinal and metabolic obesity-associated disorders. However, we still do not know which type of polyphenol are providing those beneficial effects and the underlying mechanisms.

A better understanding of the influence of specific blueberry polyphenols on the gut microbiota and intestinal epithelium is crucial to understand their role in metabolic disorders associated with obesity. We hypothesize that the gut microbiota, the host colonic-epithelium and the mucus profile are key features by which non-absorbable oligomeric and polymeric PACs may improve the perturbed intestinal and metabolic homeostasis associated with an obesogenic diet. Furthermore, the identification of bacterial communities displaying unique phenolic degrading capability, may reveal specific bacterial consortia triggering beneficial bioactivities in the host.

In the current study, we evaluated the contribution of specific polyphenolic fractions found in whole wild blueberries to their beneficial impact on intestinal and metabolic endpoints in a mouse model of diet-induced obesity. We investigated the impact of the oral administration of wild blueberry extract (hydro-ethanolic extract, WBE) or three polyphenolic fractions (anthocyanins and phenolic acids, oligomeric PACs with flavonols and phenolic acids, and PACs polymers in the respective amounts found in the whole blueberry extract) on the diet-induced glucose intolerance, insulin sensitivity and on the gut microbiota composition, as well as on intestinal epithelium histology.

## Results

### Phenolic characterization of WBE and polyphenolic fractions

**Figure 1:**
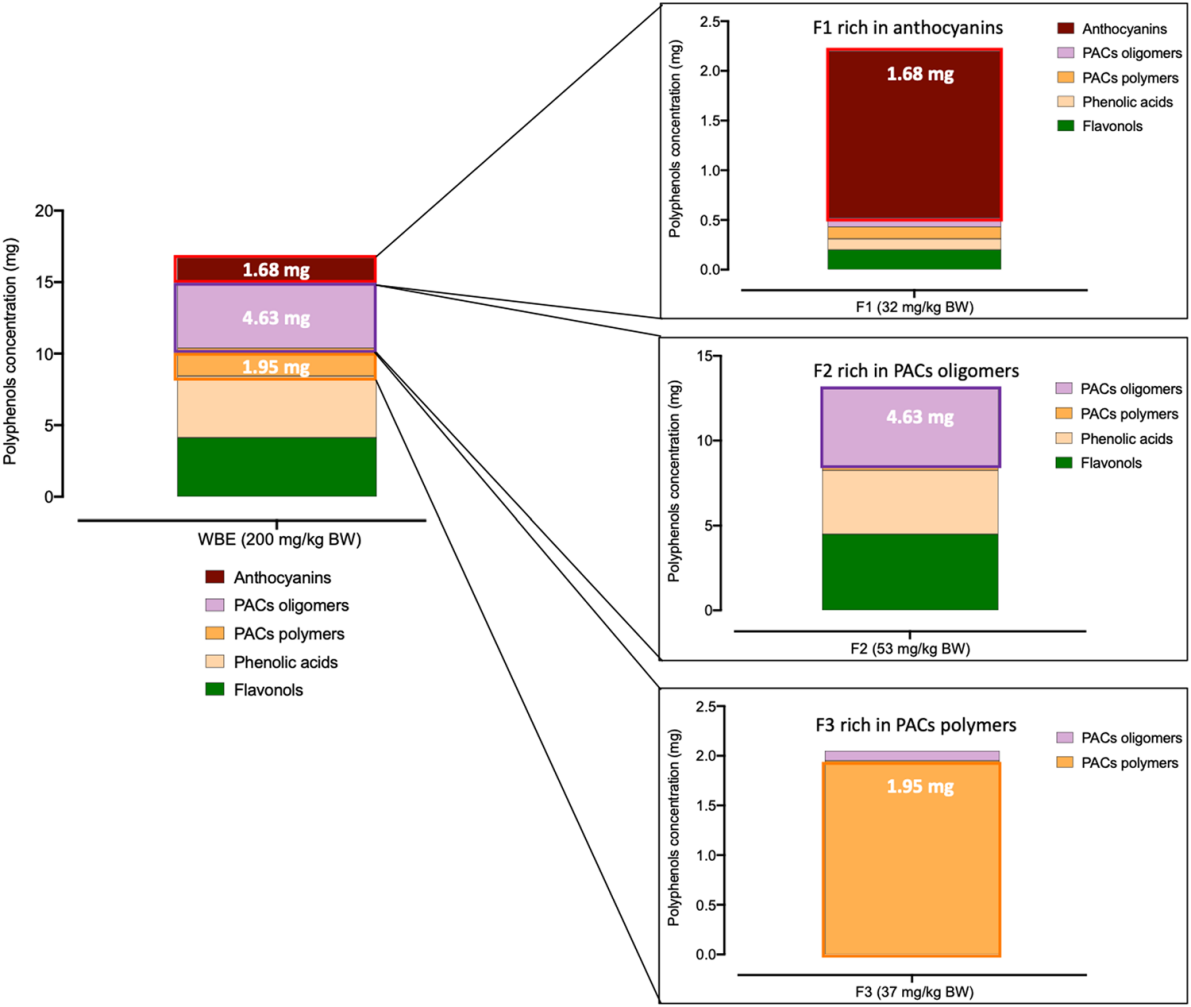
Phenolic content in mouse dosage of blueberry extract and polyphenolic fractions. C57BL/6J mice were fed a HFHS diet and treated either with the vehicle (water); 200 mg/kg body weight (BW) of whole blueberry extract (WBE) containing 17 mg of polyphenols; 37 mg/kg BW of the F1 fraction rich in anthocyanin and phenolic acids; 53 mg/kg BW of the F2 fraction rich in oligomeric PACs, phenolic acids and flavonols, and 37 mg/kg BW of the F3 fraction rich in polymeric PACs for 8 weeks. The dose of each fraction administrated to mice were calculated to provide the same concentration of anthocyanins, PACs oligomers and PACs polymers as encountered in the whole blueberry extract (F1, F2, and F3 fractions respectively).

The polyphenolic characterization of the extracts and fractions is shown in the Supplementary Information Table S1. The rationale for the fractionation of the wild blueberry polyphenolic extract (WBE) was to determine which polyphenol class and type, that is, anthocyanins, PACs oligomers or PACs polymers, was responsible for the effects observed in the whole WBE. To do so, the whole WBE was fractionated in three parts, F1) anthocyanins and phenolic acids, F2) oligomeric proanthocyanidins (PACs), phenolic acids and flavonols (PACs degree of polymerization DP < 4), and F3) PACs polymers (PACs DP > 4) (Fig. 1**)** and supplied at their respective concentration in the whole extract. That is, two hundred mg of WBE per kg mouse body weight (BW) provided a daily dose of 17.03 mg of total polyphenols, including phenolic acids, flavonols, anthocyanins, PACs oligomers and polymers. 32.01 mg/kg BW of fraction F1 administrated to mice represented the proportion of anthocyanins contained in the whole 200 mg/kg BW WBE (i.e. 1.68 mg), while 53 mg/kg BW of fraction F2 supplied the equivalent daily dose of oligomeric PACs (i.e. 4.43 mg) and 37.21 mg/kg BW of fractions F3, provided an equivalent dose of polymeric PACs supplied by the WBE (i.e. 1.95 mg). The detailed characterization of extracts dosage per mouse is presented in Fig. 1. Noteworthy, the dose used in this study in mice represents a feasible dose in humans. By applying the US Food and Drug Administration’s guidelines to establish the human equivalent dose based on body surface area^22^, we found that a 17 mg/kg dose would be the human equivalent of a 200 mg/kg dose in mice.

### Weight gain and adiposity were not affected by the WBE and BPF

As expected, the consumption of a HFHS diet for 8 weeks significantly increased mouse body weight and body weight gain as compare to the chow-fed mice (Supplementary Information Fig. S1A,B). This was explained by increased energy intake (Supplementary Information Fig. S1C). The energy intake was slightly, but significantly decreased at week 2 in the animals treated with the WBE and with fraction F2 as compared to the HFHS control group (p < 0.05) (Supplementary Information Fig. S1C). This effect was transient and no difference in total energy intake was observed for the whole duration of the experiment (HFHS, 600.7 ± 15.88 kcal; WBE, 583.2 ± 9.69 kcal; F1, 589.0 ± 12,17 kcal; F2, 577.6 ± 11.49 kcal; F3, 574.7 ± 9.06 kcal). Visceral adipose tissues were significantly increased in the HFHS control group compared to chow-fed mice (p < 0.05). However, mice gavaged with WBE and BPF showed similar visceral mass with no significant differences compared to HFHS-vehicle treated mice (Supplementary Information Fig. S1D).

### Total WBE and F3 rich in polymeric PACs improved glucose tolerance in HFHS-fed mice

**Figure 2:**
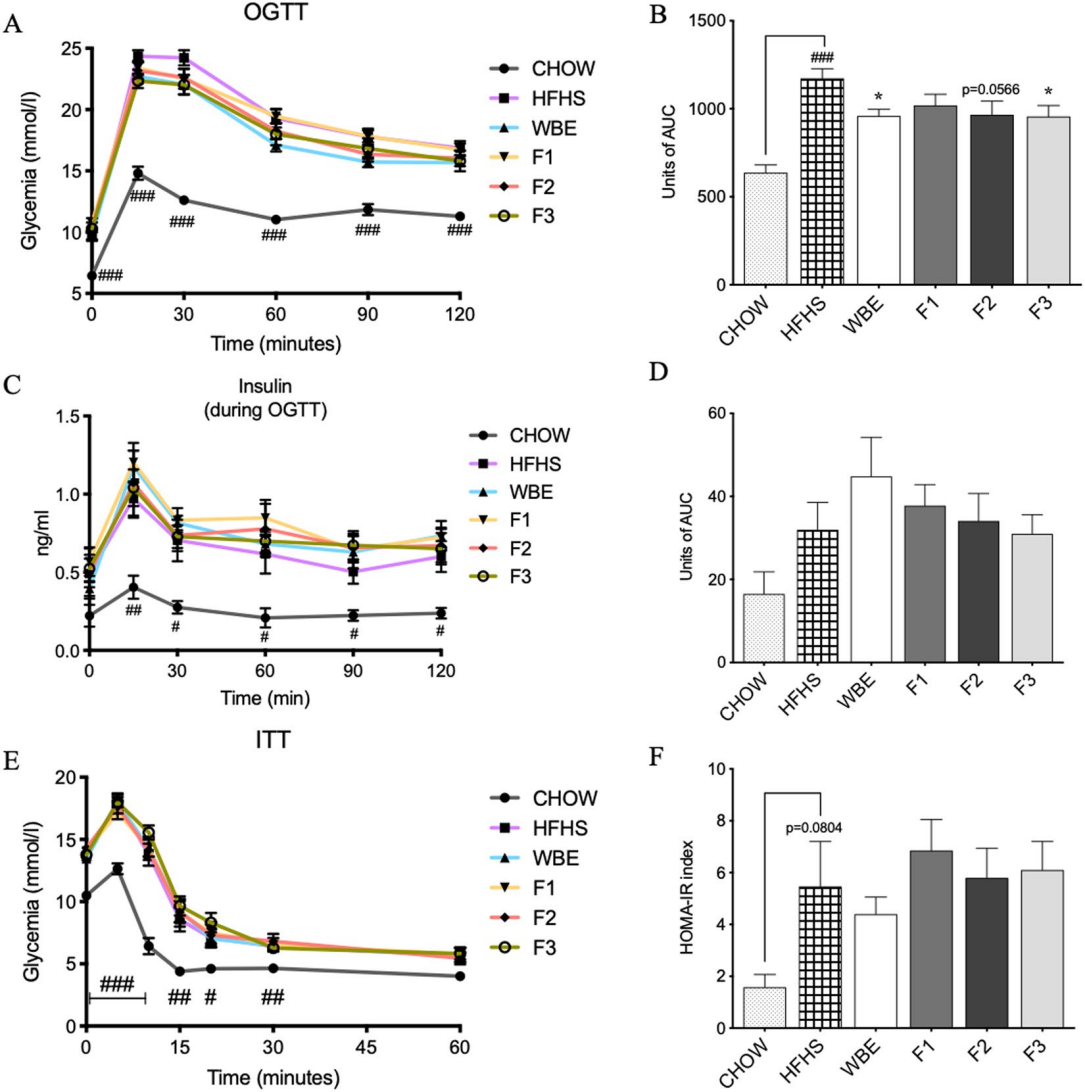
Total WBE and polymeric PACs-rich fraction fed mice presented an improved glucose tolerance. C57BL/6J mice were fed a HFHS diet and treated either with the vehicle (water), the WBE or a BPF: anthocyanin and phenolic acids (F1), oligomeric PACs, phenolic acids and flavonols (F2) and polymeric PACs (F3) for 8 weeks. (**A**) Oral glucose tolerance test (OGTT) was performed with 12 h fasted at 8 weeks of treatment; (**B**) Area under the curve (AUC) of OGTT calculated between baseline and 120 min; (**C**) Insulin levels were measured during the OGTT; (**D**) AUC of insulin level during OGTT was calculated; (**E**) Blood glucose measurements over insulin tolerance test (ITT) was performed on fasted mice for 6 h at 6 weeks of treatment; (**F**) Homeostatic model assessment of insulin resistance (HOMA-IR). A student-t test was applied to calculate the significance of the difference between the chow and the HFHS group. One-way ANOVA with a Dunnett post hoc test was applied to calculate the significant differences between groups. Two-way repeated measures ANOVA with a Dunnett post hoc test was applied to calculate the significance between groups at different time points. Values are expressed as the mean ± SEM (n = 12/groups). *p < 0.05 compared to HFHS; Chow vs HFHS ^#^p < 0,05, ^###^p < 0,001.

Further assessment of glucose homeostasis was determined by the oral glucose tolerance test (OGTT) (Fig. 2A). Glucose tolerance was markedly deteriorated in the vehicle-treated HFHS mice as revealed by the significant increase of the area under the curve (AUC) of the OGTT as compared to the chow-fed mice (p < 0.05). While fasting glycemia was not different between WBE and BPF-treated mice and the vehicle-treated HFHS group, we observed a significant improvement in glucose tolerance in mice treated with the WBE and F3 polymeric PACs as indicated by an a significant AUC reduction of 18.25 and 18.52%, respectively (p < 0.05), whereas a trend in decreased AUC (17.68%, p = 0.0566) was also observed in mice fed the F2 fraction as compared to HFHS (Fig. 2B). HFHS-fed mice were insulin-resistant compared to chow-fed mice as indicated by the elevated glucose-stimulated insulin secretion (GSIS) (Fig. 2C,D**)**, the impaired insulin tolerance (Fig. 2E) and the HOMA-IR index (Fig. 2F). However, no significant effect on the above parameters was observed in mice fed the WBE and BPF compared to HFHS-fed group.

### The polymeric PACs fraction (F3) increased the total GC number in HFHS-fed mice

**Figure 3:**
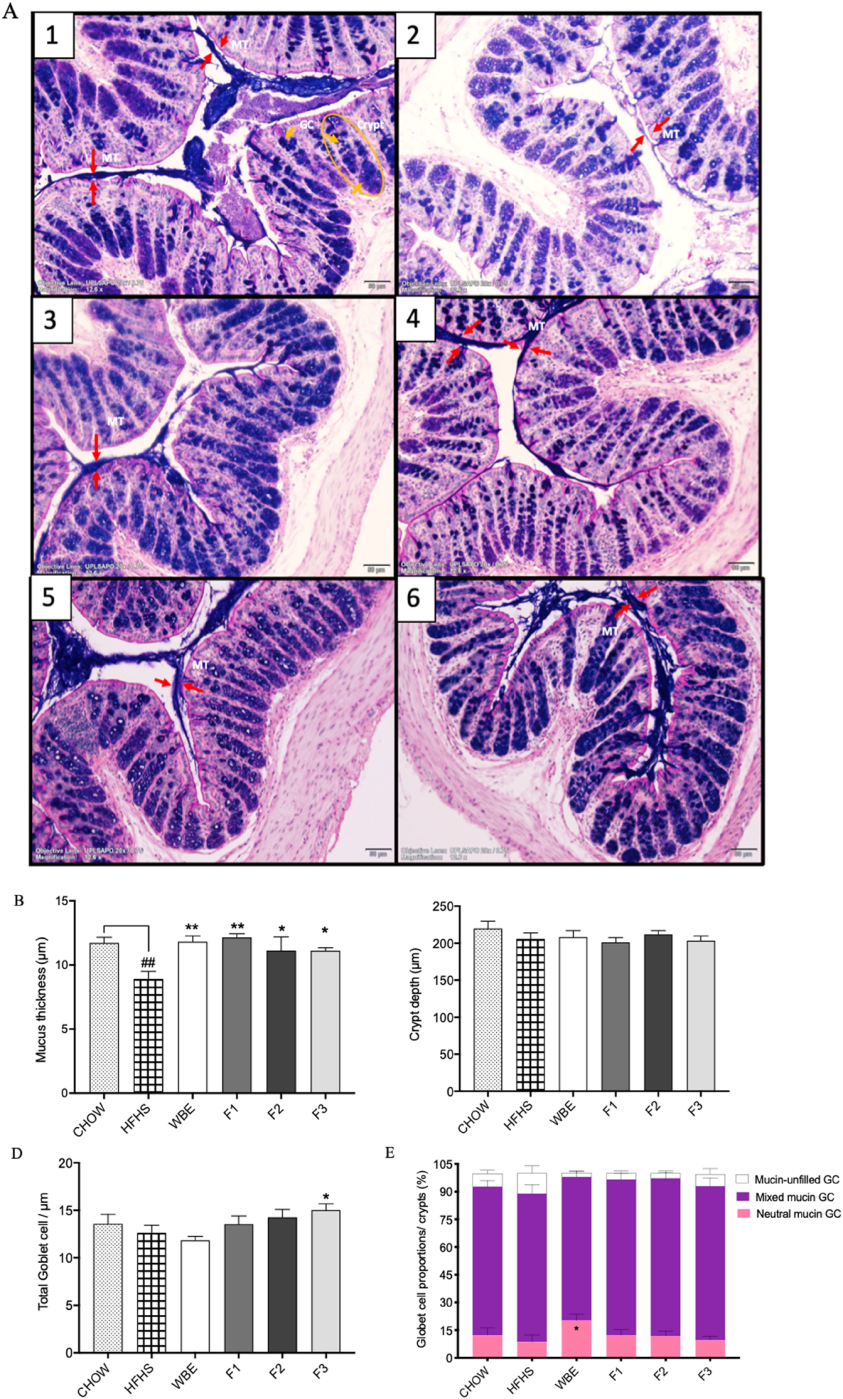
Wild blueberry polyphenolic fractions restore the mucus thickness and polymeric PACs-rich fraction significantly stimulated mucin-secreting goblet cells number in HFHS-fed mouse colon. The effect of HFHS-diet and the supplementation with WBE and BPF: anthocyanin and phenolic acids (F1), oligomeric PACs, phenolic acids and flavonols (F2) and polymeric PACs (F3) were studied in mouse colon morphology. A combination of Alcian Blue and periodic acid–Schiff staining (AB/PAS) was used to distinguish acidic (dark blue) and neutral (red) mucins. A purple color indicates the presence of both acidic and neutral mucins. Images shown are representatives of examined mice (n = 12) in each group: (**A-**1) Chow; (**A-**2) HFHS; (**A-**3) WBE; (**A-**4) F1; (**A-**5) F2; (**A-**6) F3. Images were taken using objective lens UPLSAPO 20×/0.75, magnification 12.6×, Scale 50 μm. Arrows indicate mucus thickness (MT) measured using Image J software. The circle is enclosing a colonic crypt. Within each crypt mucin-filled goblet cells (GC) were counted. Histological parameters were evaluated in cross-sections of colon tissues stained with AB/PAS staining: (**b**) Mucus thickness, (**c**) Crypt depth (**d**) Total GC number per µm of crypt, and (**e**) Mucin-filled GC types. Data are shown as Mean ± SEM (n = 12/group). Significant differences were determined by ordinary one-way ANOVA. Values are expressed as mean +/− SEM. p < 0.01^##^ compared to Chow p < 0.01** and p < 0.05* compared to HFHS, respectively.

The intestinal mucus layer has important protective and lubricative properties in the colon and may be affected by a HFHS-diet induced dysbiosis, thereby augmenting the risk of colonic mucosa exposition to the commensal microbiota. This prompted us to investigate the effects of wild blueberry polyphenols on mouse colon mucosal histomorphology, including the crypts length, the mucin secreting goblet cells (GC) number and the mucus layer thickness. Representative images of AB-PAS stained colonic tissues are presented in the Fig. 3A1–6. In chow-fed mice, a thick purple stained layer of mucus was consistently observed, with inner mucus layer closely associated to the colonic epithelium and a secreted mucus scattered in the luminal space. Mucus layer was thinner and sparse in the vehicle-treated HFHS group (Fig. 3A-2) as compared to the chow-fed animals. However, the WBE and BPF-treated mice displayed a restored mucus thickness as compared to the HFHS control group (Fig. 3A3–6). HFHS-fed mice presented a thinner mucus layer compared to the Chow-fed mice (p < 0.01, Fig. 3B). However, all mice orally supplemented with either WBE- (p < 0.01), F1- (p < 0.01), F2- (p < 0.05) or F3 had a significantly thicker mucus layer as compared to the vehicle-treated HFHS-fed mice (p < 0.05). No difference in the crypt’s depth was observed among the WBE and all the BPF-treated mice and vehicle-treated HFHS control mice (Fig. 3C). Although we observed that the mucus layer in the vehicle-treated HFHS-fed mice was thinner as compared to the chow group, there was no significant differences in the number of total mucin-secreting GC. Once normalized over the length of the crypt (GC number per μm of colonic crypts), only mice fed the F3 fraction rich in polymeric PACs presented an increased number of total GC compared to vehicle treated HFHS fed mice (p < 0.05) (Fig. 3D).

Consistent with the multiple functions of mucus, it is well established that GC produce a heterogeneous range of secretory mucins subtypes^23^ and can be identified as acid, neutral and as a mix of both mucins. The acid mucin are further differentiated into sialomucins or sulfomucins groups, based on the presence of terminal sialic acid or sulfate group on the O-linked oligosaccharide chains^14^. Therefore, we classified the total number of GC considering the polychromatic AB/PAS mucin type separately, resulting in the specific determination of acid mucin-filled GC, mixed mucin-filled GC, and neutral mucin-filled GC, as well as, those GC without mucin that were considered as mucin empty GC. A mix of neutral and acidic mucins (purple stained mucin, mixed mucin GC) was predominantly found in colonic GC in BPF-treated groups (F1, 84.2%; F2, 85.3%; and F3, 83.4%; compared to vehicle-treated HFHS-fed mice, 80.2%) (Fig. 3E). However, in mice fed WBE, the neutral mucin-filled GC showed an increased proportion (20.2%) compared to HFHS-fed mice (8.6%, p < 0.05) (Fig. 3E). In contrast, the remaining GC in HFHS-fed mice had a higher proportion of mucin-unfilled GC (11%) compared to the WBE (2.2%) (p = 0.0358, q = 0.093 after multiple comparison corrections), and BPF treated mice (F1, 3.3%; F2. 2.7%; and F3, 6.4%) (F1, p = 0.0536 q = 0.0938; F2, p = 0.0338 q = 0.093).

To investigate the influence of wild blueberry polyphenols on intestinal permeability *Tjp1* (Tight junction protein, also known as Zonula occludin, ZO-1) and *Ocln* (Occludin) gene expression were measured using RT-qPCR and found no significant changes between vehicle treated HFHS and chow-fed mice as compared to mice treated with WBE and BPF (Fig. S2A,B).

### WBE and BPF did not affect the microbial richness of the gut microbiota of HFHS-diet induced obese mice

To determine whether HFHS-diet affected fecal microbial diversity and whether the blueberry polyphenol treatments could modify this parameter, we next analyzed alpha and beta diversity indices. Rarefaction curves of the normalized sequences showed that our sequencing was deep enough to include all of the OTUs in the diet groups (Fig. S3). All treatments, including vehicle-treated chow and HFHS fed mice exhibited nearly the same α-diversity index using the *Chao1* index, an estimate of OTU richness for microbial communities (Fig. S3A). There were no significant differences in the number of observed species in the polyphenols-treated mice compared to the HFHS-fed mice, as determined by Kruskal-Wallis test with Benjamini multiple comparison correction. A similar response was observed between the BPF-treated mice and the HFHS-fed mice on species abundance and Shannon’s diversity index (*P* > 0.05) (Fig. S3B).

**Figure 4:**
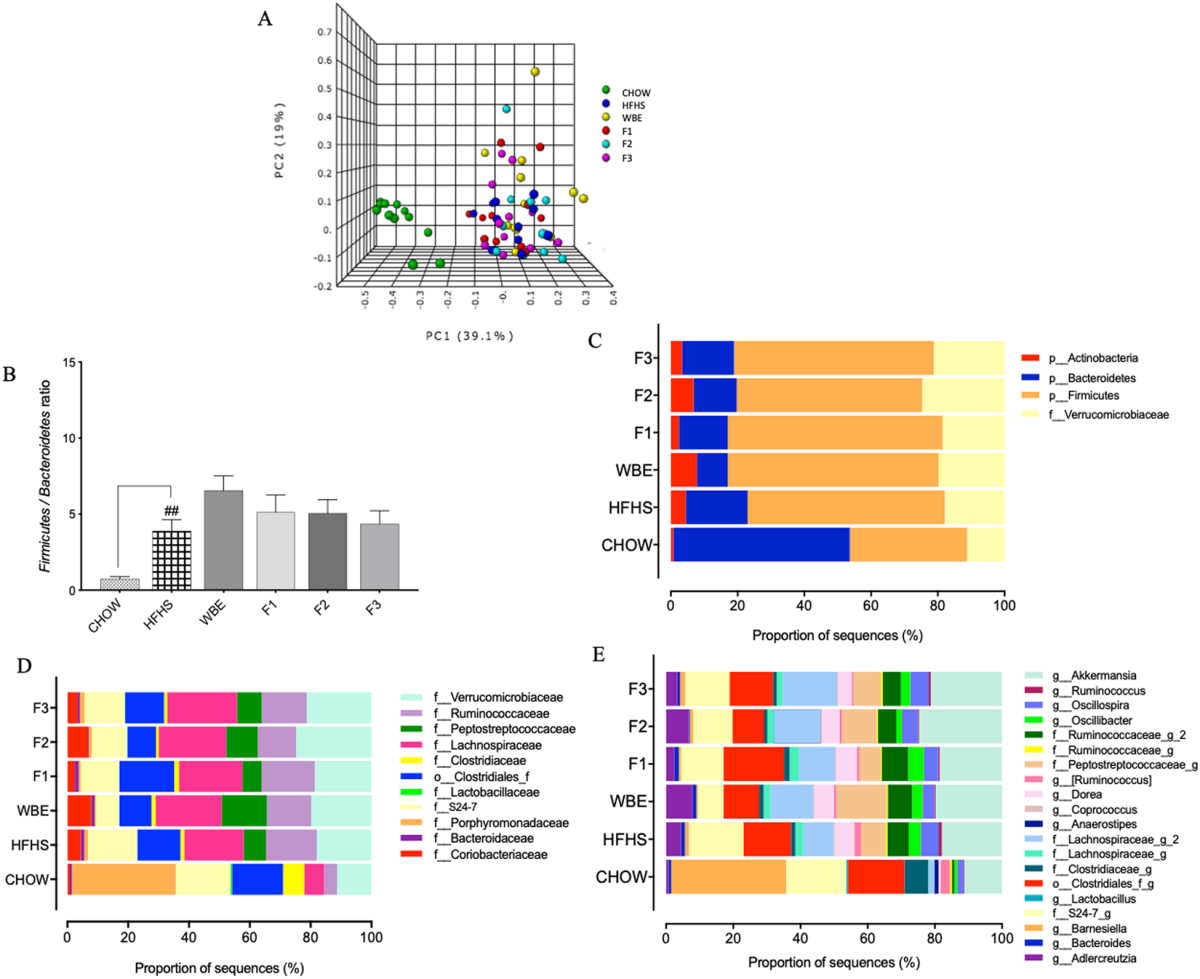
Dissimilarity analysis of the gut microbiota composition between groups and the mean relative abundance of bacterial taxonomies at phylum, family and genus level after 8 weeks of dietary supplementation. Principal coordinate (PCoA) plots exhibited a different dispersion of WBE fecal microbial communities. Quantitative non-phylogenetic measures were applied as β-diversity metrics to calculate distances among all fecal mouse samples. (**a**) Bray Curtis matrix of samples rarefied to 6,957 sequences revealed significant differences between groups (PERMANOVA, R-squared: 0.37968; p-value < 0.001). Each sample point (n = 12/group) is color-coded based on the administrated diet for 8 weeks (W8), as shown in each figure legend. (**b**) Different *Firmicutes* to *Bacteroides* ratio are shown for Chow and HFHS-fed controls mice at week 8. Mice fed HFHS-diet and supplemented with WBE and BPF: anthocyanin and phenolic acids (F1), oligomeric PACs, phenolic acids and flavonols (F2) and polymeric PACs (F3) showed variations on the proportions of sequences distributed in (**c**) Four bacterial phyla, (**d**) Eleven families and (**e**) twenty assigned genus. The statistical significance on β-diversity across sample groups was assessed with the non-parametric Permutational Multivariate Analysis of Variance (PERMANOVA, 999 Monte Carlo permutations) test. Kruskal-Wallis test with Benjamini post-hoc multiple comparison correction compared to HFHS were used to found out significant changes in the taxonomic proportions, p < 0.01^##^. Values are expressed as mean +/− SEM.

As expected, the HFHS-diet drastically changed the gut microbiota composition compared to the chow-diet. PERMANOVA for the Bray Curtis matrix revealed significant differences between vehicle-treated chow and HFHS and BPF-treated groups (PERMANOVA, R-squared: 0.37968; p-value < 0.001). The Bray Curtis β-diversity index further showed that the communities associated with the chow-group and the vehicle-treated HFHS-fed mice diverged upon a confined region within the PCoA **(**Fig. 4A). Specifically, the distribution of mice across the PC1 axis reflected the difference of microbial profile between mice fed chow vs HFHS diets, explaining 39% of the variance. Although there were differences in the relative proportion of some identified bacterial OTUs, the β-diversity of the gut microbiota remained similar between the mice fed the polyphenols-containing diets. The mice fed the WBE, the BPF and the control HFHS-diet clustered similarly, this was not the case for mice fed the WBE who tended to spread out of the vehicle-treated HFHS-group.

### Bacterial taxa within the families *Coriobacteriaceae* and *Verrucomicrobiaceae* were influenced by the WBE and the F2 fraction rich in oligomeric PACs in HFHS-diet induced obese mice

**Figure 5:**
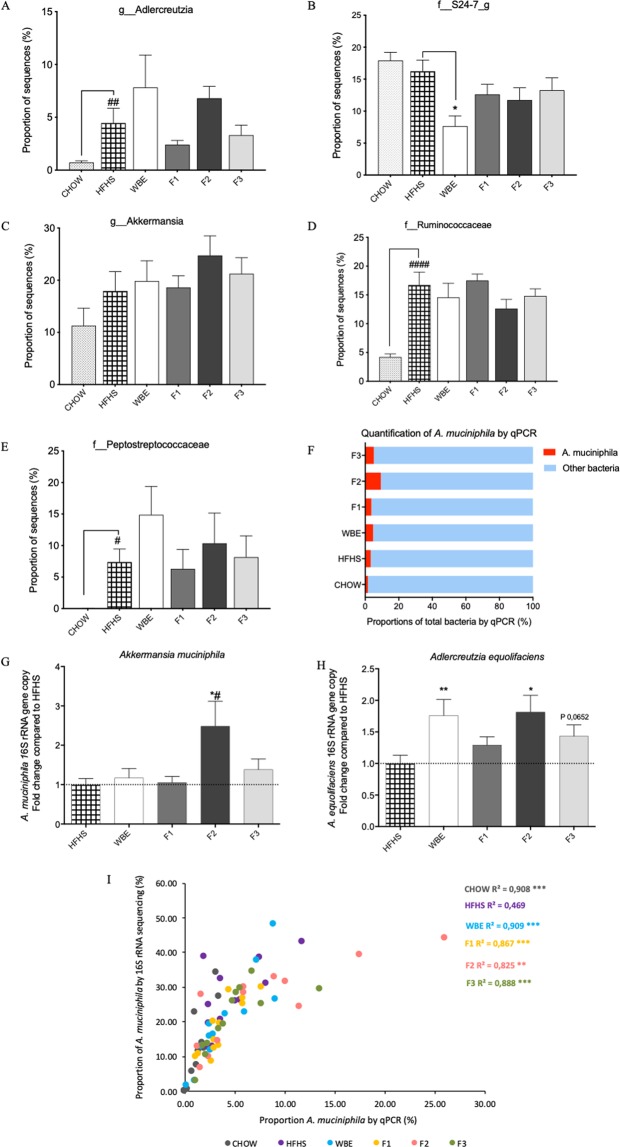
Mean proportions of bacterial taxa by 16S rRNA gene sequencing and significant differences in relative mean proportion of *A*. *muciniphila* and *A*. *equolifaciens* by qPCR on blueberry polyphenols treated mice and HFHS control group. HFHS-fed mouse fecal samples were analyzed by 16S rRNA gene sequencing and qPCR analysis of 16S rRNA gene copy for *Adlercreutzia equolifaciens* and *Akkermansia muciniphila* were performed to validate the data obtained by high-throughput 16S rRNA sequencing method after 8 weeks of dietary treatment with WBE and BPF: anthocyanin and phenolic acids (F1), oligomeric PACs, phenolic acids and flavonols (F2) and polymeric PACs (F3). Relative proportion of sequences of (**a**) *Adlercreutzia equolifaciens*, (**b**) An unassigned genus of the family *S24-7* and (**c**) *Akkermansia muciniphila*; the relative proportion of 16S rRNA gene copy determined by qPCR of (**d**) An unassigned genus of the family *Ruminococcaceae*; (**e**) An unassigned genus of the family *Peptostreptococcaceae*; (**f**) The relative proportion of *Akkermansia muciniphila* relative to the total bacteria; (**g**) Fold-change of the *Akkermansia muciniphila* proportion relative to HFHS (**h**) Fold-change of the *Adlercreutzia equolifaciens* proportion relative to HFHS; (**i**) Spearman correlations analysis was performed for comparing the data obtained from 16S rRNA sequencing versus the 16S rRNA relative proportion obtained by qPCR, Statistic: *p < 0.05, **p < 0.01, ***p < 0.001 for qPCR vs 16S rRNA gene sequencing data. Values are expressed as mean +/− SEM. Kruskal-Wallis test with Benjamini post-hoc multiple comparison correction compared to HFHS *p < 0.05 and **p < 0.01. Mann-Whitney U-Test PACs < 4 vs HFHS ^#^p < 0.05.

Analysis of bacterial relative abundance confirmed prior reports^24^ that the consumption of a HFHS diet consistently affects the proportion of *Bacteroidetes* (19%) and *Firmicutes* (59%), compared to a chow diet (53% and 35%, respectively) (p < 0.001), leading to a higher *Firmicutes/Bacteroidetes* ratio (*F/B*) in the HFHS treated groups (Fig. 4B,C). Phylum level analysis showed that most of the compositional differences between vehicle-treated HFHS-fed mice, WBE and BPF treated mice were reflected in the relative abundance of *Firmicutes*, *Bacteroidetes* and, to a lesser extent, *Verrucomicrobia* and *Actinobacteria* **(**Fig. 4C). In the WBE and BPF treated mice, the family *Coriobacteriaceae*, *S24-7*, *Verrucomicrobia* and the order *Clostridiales* were favored as compared to HFHS (Fig. 4D). Within the families, 20 different genera were identified (Fig. 4E). Important changes in the relative proportion of the genera *Adlercreutzia*, *Akkermansia*, an unknown genus of order *Clostridiales*, family *S24-7*, as well as a genus of *Ruminococcaceae* and *Peptostreptococcaceae* were observed (Fig. 5A–E**)**. Kruskal-Wallis tests with Benjamini’s multiple corrections showed a significant reduction of an unassigned genus of the family *S24-7* by WBE compared to vehicle-treated HFHS-group (p < 0.05). Although it was not supported statistically, we noted that BPF tended to increase *Akkermansia* (family *Verrucomicrobiaceae*) abundance, presenting the higher proportion in the mice treated by the F2 fraction rich in oligomeric PACs (24.8%) and the F3 rich in polymeric PACs (21.2%) compared to HFHS (17.9%). The WBE did not stimulate *A*. *muciniphila*. Moreover, unassigned genera belonging to the families *Ruminococcaceae* and *Peptostreptocaccaceae* were increased in WBE and reduced in F2 treated mice (Fig. 5D,E). Similarly, an increased proportion of *Adlercreutzia* (family *Coriobacteriaceae*) was observed in the WBE (7.8%) and F2-fed mice (6.8%) compared to HFHS-fed mice (4.5%).

### qPCR analyses of mouse fecal samples confirmed the stimulatory effects of WBE and the F2 fraction rich in oligomeric PACs on the relative proportion of *A*. *muciniphila* and *A*. *equolifaciens*

The relative abundance of distinctive bacterial genus found in the 16S rRNA data sequencing by the BPF were further analyzed by qPCR. We focused our attention on two bacterial species considered important for the degradation of polyphenols and the attenuation of metabolic issues; *Adlercreutzia equolifaciens*, and *Akkemansia muciniphila*. Interestingly, *A*. *muciniphila* proportion was significantly stimulated by the F2 fraction rich in oligomeric PACs, showing a 2.5-fold higher relative proportion of the bacterium in the feces of those mice as compared to that of the HFHS group (p < 0.05). At the same time, the WBE, which contained similar amount of oligomeric PAC, did not stimulate *A*. *muciniphila* (Fig. 5F,G). In a similar way, the *A*. *equolifaciens* relative proportion was 2.0-fold higher in both mouse groups treated with the WBE and the F2 fraction (p < 0.01 and p < 0.05 respectively) (Fig. 5H). Both qPCR and 16S rRNA gene sequencing sets of data were correlated in order to validate the observed abundance. Significant spearman coefficients were obtained for the groups (p < 0.01) and showed comparable profile of those bacterial taxa by the two quantification methods (Fig. 5I**)**.

### Functional analysis of microbial communities within wild blueberry polyphenols fed mice revealed pathways related to xenobiotic metabolism

LEfSe analysis of PICRUSt predictions in vehicle-treated HFHS-fed mice demonstrated fluctuations in the functional pathways attributed to carbohydrate, lipid, and energy metabolism and membrane transport as compared to chow-fed mice. In total, 21 pathways were significantly affected by the HFHS-diet, compared to WBE supplementation, which were shown to affect only 6 pathways (Supplementary information Fig. S5). The WBE repressed microbial pathways were linked to LPS biosynthesis and glycosaminoglycan degradation in HFHS-fed mice, while WBE-fed mice revealed changes in xenobiotics metabolic pathways like benzoate degradation. Noteworthy is the fact that some functions linked to xenobiotics metabolism and cell motility stood out in mice treated with the BPF. Among them, seleno-compound metabolism, butanoate metabolism and benzoate degradation are pathways highly associated to phytochemical metabolism, such as polyphenols^25^. Those results are consistent to the increased abundance of the polyphenols-degrading family *Coriobacteriaceae*. However, the F1-treated group was the only one that revealed starch and sucrose metabolism as important feature included within the category of carbohydrate metabolism. In the case of F2 fraction rich in oligomeric PACs, the butanoate metabolism pathway was significantly overrepresented and for the F3 polymeric PACs group, the ketone body’s metabolism and plant-pathogens interaction pathways were induced. Those predicted functional changes induced by PACs suggest adaptation of the gut microbial environmental that are likely related to microbial energy metabolism.

## Discussion

The purpose of this study was to identify blueberry polyphenolic fractions that can contribute to the reported beneficial effects of the WBE on metabolic health, the gut microbiota composition and on the intestinal histology of HFHS-diet induced obese mice. We observed that WBE improvements of glucose tolerance coincided with a modulation of the abundance of bacterial families such as *Coriobacteriaceae* and *Verrucomicrobiaceae* and the maintenance of the colonic mucus layer in a murine model of diet-induced obesity and insulin resistance. We also provide new evidence that this effect is largely attributable to the blueberry proanthocyanidin polymers enriched fraction.

Indeed, within the BPF, the F3 fraction rich in polymeric PACs appears to contribute the most to the overall beneficial effect of the wild blueberry extract on intestinal histology and glucose tolerance of HFHS-fed mice, while the F2 fraction, rich in PACs oligomers, also tended to ameliorate this last parameter (p = 0.0566). Notwithstanding the fact that PACs polymers represented only a small fraction of the total polyphenols found in the whole WBE, they exerted significant effects on glucose tolerance. During the OGTT, despite no significant changes in insulin secretion in PAC treated animals as compared to the control group, an improved glucose tolerance was observed. These results may suggest an amelioration of the insulin action, an insulin independent effect or a reduced intestinal glucose absorption in the PAC treated group. Indeed, previous studies reported that polyphenols may interfere with SGLT1 and GLUT2 in the small intestine. In this regard, proanthocyanidins have been shown to mimic insulin effects by affecting intestinal glucose transporters^26^ and stimulating intestinal hormones which are involved in the modulation of digestion and metabolism^27^. For instance, Casanova-Martí *et al*.^27^ demonstrated, both *in vivo* and *ex vivo*, the role of grape seed proanthocyanidins (GSPE) on the induction of enterohormones secretion in different parts of the intestine, leading to glucose homeostasis regulation. The authors showed that GSPE treatment increased GLP-1 levels in the ileum, while the unabsorbed GSPE’s and phenolic microbial metabolites did so in the colon. These findings suggest that unabsorbed polyphenols with high degree of polymerization and microbiota-metabolized phenolic compounds act on enteroendocrine L-cells to promote GLP-1 secretion^27^. Further studies will be needed to determine whether the protection from glucose intolerance that are linked to unabsorbed polymeric PACs from blueberry can be linked to other intestinal targets that are linked to pancreatic beta-cell insulin secretion or hepatic insulin clearance.

In this study, the improved glucose tolerance was found to be independent of changes in body weight, consistent with previous studies^28–30^. For instance, Seymour *et al*.^30^ did not observe any change in body weight gain, lean and fat mass despite improved glucose tolerance in obese Zucker rats fed a high-fat diet (HFD) supplemented with 2% of blueberry powder. Also, Elks *et al*.^29^ found that blueberry powder supplementation (4%) did not affect body weight gain, but significantly attenuated the glucose intolerance and hepatic steatosis in HFD fed obese postmenopausal female mice.

A recent meta-analysis of human gut microbes associated with obesity shows weak or non-significant associations of particular taxa or overall diversity with the body mass index^31^, but it is becoming apparent that polyphenolic compounds can distinctly reshape gut bacteria and exert local and systemic protective effects against HFD-induced metabolic dysfunctions. We observed that blueberry polyphenols had no effect on the *F/B* ratio, as a marker of diet-induced gut dysbiosis^24^; however, we observed a stimulation of key bacterial species such as *A*. *muciniphila* and *A*. *equolifaciens* belonging to *Verrucomicrobia* and *Actinobacteria* phyla, respectively, in mice fed the WBE and the F2 fraction. In mice fed the WBE, a decrease in the relative abundance of *S24-7* (taxonomically classified as *Muribaculaceae*) was coherent with the reduced *Bacteroidetes* phylum abundance. The *S24-7*, is a bacterial family believed to be involved in the degradation of plant glycan, host glycan, and α-glucan carbohydrates^32^, but within the gut microbiota composition of C57-BL6 mice, it was shown to be sensitive to the administration of antibiotics^33^. Thus, we surmise that the decreased abundances of *S24-7* taxon, is the result of the antibacterial action of the total WBE^34^.

Changes in the gut microbiota have been proposed as an important mechanism of action of poorly absorbed high molecular weight polyphenols against obesity-associated metabolic disorders. The blooming of *A*. *muciniphila* by the oligomeric PACs-rich F2 fraction is particularly interesting since it was shown to drive colonic epithelial immunomodulatory response and to protect against metabolic disorders^17,35–37^. Similar effects on *A*. *muciniphila* abundance were also triggered by an ellagitannin-rich extract from pomegranate^38^ and camu-camu^39^, by a B-type proanthocyanins-rich grape extract^40^, by an A-type proanthocyanins-rich cranberry extract^17^, as well as by lingonberries^41^. In the present experiment, there was no association of this bacterium with body weight gain. Likewise, Shin *et al*.^37^ demonstrated that blooming of *A*. *muciniphila* upon metformin treatment did not affect body weight gain or fat mass accumulation, but did improve glucose tolerance in HFD-fed mice. A recent study demonstrated that the administration of *A*. *muciniphila* counteracted diet-induced metabolic endotoxemia, restored colon mucosal barrier dysfunction and glucose homeostasis in obese type 2 diabetic mice^36^. In our study, the changes induced by the F2 fraction on the gut microbiota, and particularly on *A*. *muciniphila*, while compelling, are somewhat surprising, since they were not reproduced in the WBE treatment containing the same polyphenols as F2 (PAC oligomers, phenolic acids such as protocatechuic acid and 5- caffeoylquinic acid, as well as flavonols such as quercetin). We surmise that the polyphenols not present in F2, namely anthocyanin and PAC polymers have blunted the *Akkermansia* bloom in the WBE treated mice, probably by stimulating the growth of bacteria competing with *A*. *muciniphila*. We indeed observed that the mucus associated bacterial families, *Ruminococcaceae* and *Peptostreptococcaceae* were stimulated in the WBE and reduced in F2 treated mice (Fig. 5D,E). These species may be mutually co-exclusive to *Akkermansia* as shown by Lavelle *et al*.^42^ in a human ulcerative colitis human model^42^.

Noteworthy, the F2 fraction also favored *A*. *equolifaciens* belonging to the family *Coriobacteriaceae*, involved in the degradation of phenolic compounds including flavan-3-ols^43,44^, a feature that explains its increased relative proportion in WBE and PACs treatments compared to HFHS-vehicle treated mice. Recently, Jiao *et al*.^18^ found that *Adlercreutzia* was prevalent in low fat diet-fed mice compared to high-fat diet-induced obesity in C57BL/6J mice, and that the supplementation with blueberry polyphenolic extract increased its relative abundance. Interestingly, recent studies have not only demonstrated *Adlercreutzia*’s capacity to convert – epigallocatechin (−EGC), -epicatechin (−EC), -catechin (−C), and +catechin (+C) into their corresponding metabolites, but also that it can catalyze the above metabolites by hydroxylation reactions^44^ and produce a range of phenyl-valerolactones metabolites presumed to have metabolic bioactivities^45^. Moreover, *A*. *equolifaciens* has also been observed to be decreased in subjects suffering of inflammatory bowel disease (IBD)^46^. In that study, *A*. *equolifaciens* was considered a microbial biomarker of the reestablishment of mucosal function in those patients who positively responded to an immunomodulators monotherapy^46^.

Directly involved in intestinal homeostasis, the mucus layer covering the colonic epithelium acts as a protective barrier against the luminal commensal microbes. We observed that the supplementation with the total WBE and all the BPF prevented the loss of mucus thickness that was induced by the HFHS-rich diet. Interestingly, only the mice fed the F3 fraction rich in PACs polymers presented a higher number of colonic mucin-secreting GC on the epithelium as compared to HFHS-fed mice. Our results are comparable to those of another study which examined how cranberry PACs administration counteracted the altered intestinal morphology and function, induced by elemental enteral nutrition in mice^47^. Although in the present study the *Muc2* gene expression was not analyzed, it is well known that the mucin exocytosis and mucus function is largely regulated at the post-translational level and therefore, the analysis of *Muc2* protein content by using an *ex vivo* technique, histological or by immunostaining preparations in future studies will be useful to provide complementary evidence of improved mucus function and the potential mechanisms involved at the molecular level^48^. The histological assessment of mouse colonic tissues showed a clear improvement of the mucus barrier by the BPF, however, the innate immune pathways involved in the regulation and exocytosis of mucin remain to be explored to further understand the mechanisms underlying the enhanced colonic mucus thickness. For instance, the NLRP6-inflammasome pathway has been shown as an essential factor for mucosal self-renewal, cell proliferation, and regulation of intestinal flora through mucus secretion from GC and the epithelium^49^. Interestingly, a recent study demonstrated that a freeze-dried powder of a polyphenol-rich mulberry juice promotes the formation of NLRP6 inflammasomes and provides mucosal protection by modifying the bacterial content and preserving the GC number in mice with DSS-induced acute colitis^50^.

The type of secreted mucins is an important parameter that can be affected in intestinal metabolic disorders^51^. The proportions between neutral and acid mucins are normally constant and are modified in obesity and IBD^14^. In our experiment, significant differences were observed in the proportion of neutral mucin-filled GC between the WBE-treated (20.2%) and HFHS-fed mice (8.6%). Neutral mucins have usually been associated with mono-sulfated mucin in GC residing on the colonic crypt bottom^14^, a location where cell divisions and mucin maturation appear to be active^52^. The GC located in the top of colonic crypt primarily harbor acid-sialomucins^14^. In the case of the BPF-treated mice, a trend toward increased acidic-mixed mucin was observed, suggesting that BPF may induce the secretion of sialomucins which are more resistant against microbiological degradation than neutral mucins and are impervious to bacterial glycosidases and host proteases^53^. Similar mucin profile has been reported in normal human colonic tissues, with a predominance of acidic-mixed mucin (~80%) and sparse neutral mucin (20%)^54^. These results highlight the modulation of maturation and mucin-type profile as an emerging mechanism through which wild blueberry polyphenol may maintain intestinal homeostasis.

One limitation of the present study is that our methodological approach for the preparation of polyphenolic fractions did not permit the obtention of pure phenolic classes, as it was the case for the anthocyanins rich fraction (F1) and the oligomeric PACs rich fraction (F2). To confirm the effects of WBE and the BPF, in particular, an improved fractionation of wild blueberry polyphenols will be required in order to test the selective effects of PAC oligomers and polymers on the gut microbiota composition (*Akkermansia* and *Adlercreutzia*) and histology, as well as on the related metabolic phenotypes in further studies.

In summary, we demonstrated that the oral administration of all BPF reestablished the colonic mucus thickness in obese mice and thus created an ecological niche for the symbiotic mucosa-associated bacteria. Specifically, the F2 fraction exerted a selective prebiotic action on the mucin-degrading bacterium *A*. *muciniphila*, but this was not the case with the WBE. Interestingly, we also observed a bloom of the polyphenols-degrading family *Coriobacteriaceae*, in particular of *A*. *equolifaciens*, which suggest their involvement in the metabolism of polyphenols, which is particularly relevant as it can generate bioactive molecules involved into the amelioration of metabolic disturbances in obesity and diabetes^55^. Our study also unraveled the important role that polymeric PACs play on the gut epithelial barrier function, especially promoting a thicker mucus layer, possibly through increasing mucin-secreting GC number, as well as mediating non-insulin dependent intestinal glucose response in obese mice. While the administration of WBE attenuated HFHS-induced glucose tolerance, only the F3 fraction replicated this effect, even though this fraction was in lower proportion in the whole WBE.

## Methods

### Polyphenols fractionation

A polyphenol-rich wild blueberry hydro-ethanolic extract (70%) *(Vaccinium angustifolium* Aiton) (WBE) provided by Diana Food Canada was fractionated by gel-filtration chromatography as described by Gu *et al*.^56^ to produce three different fractions, the first, rich in anthocyanins and phenolic acids (F1), the second, rich in PACs oligomers, phenolic acids and flavonols (F2) and the last one, rich in PACs polymers (F3). The purification of anthocyanin compounds was carried out using 30 g of cation exchange resin (siliaPrepX SCX, silicycle, Canada). Briefly, 75 g/l of crude WBE solubilized in acidified water (1% acetic acid) was deposited on the resin, and the WBE was eluted with 50% ethanol (Fraction containing flavonoids and PACs), and further with acidified methanol with 5% of HCl (F1 containing anthocyanins). Anthocyanins-containing fraction was then passed through XAD 7 resin in order to eliminate the HCl that may be toxic for mice and further evaporated and lyophilized. The fraction containing flavonoids and PACs was also evaporated and recovered in Ethanol/water solution (50/50: v/v). The purification of the PACs-rich fraction was further performed on a Sephadex LH-20 column using an adaptation of the protocol of Feliciano *et al*.^57^. Redisep column (Teledyne, Isco) containing 65 g of Sephadex LH-20 (bed volume = 100 ml) was placed on a Spot Prep system (Armen Instrument, France). The system is coupled to a UV detector set at 254 nm, and a fluorescence detector (emission wavelength: 230 nm – reception wavelength: 321 nm). The flow rate used was 5 ml/min. PACs oligomers and flavonoids were isolated by elution with methanol/ethanol 50/50 (F2); and the PACs polymers were eluted with 70% acetone (F3). In order to produce the needed amount for the preclinical study, several consecutive separations were carried out. The fractions were then homogenized and characterized.

### Phenolic characterization of blueberry extract and polyphenolic fractions

The polyphenol composition analysis was carried out as described previously^58^. Flavonols, flavan-3-ols and phenolic acids were analyzed as published before^58^ using reverse-phase UHPLC–MS/MS, where data were acquired in scan mode (m/z 100–1000). Anthocyanins were analyzed by reverse-phase HPLC with DAD detection using an Agilent 1100 series system (Santa Clara, CA). The separation was performed with a flow rate of 1 mL/min using a Develosil C18 reverse phase column (250 mm × 4 mm, 5 μm particle size), protected with an Ultrasep C18 guard column (Phenomenex, CA). Binary gradient of 5% formic acid in ultrapure water (solvent A) and methanol (solvent B) was as follows for the anthocyanins separation: 0–2 min, 5% B; 2–10 min, 5–20% B; 10–15 min, 20% B; 15–30 min, 20–25% B; 30–35 min, 25% B; 35–50 min, 25–33% B; 50–55 min, 33% B; 55–65 min, 33–36% B; 65–70 min, 36–45% B; 70–75 min, 45–53% B; 75–80 min, 53–55% B; 80–84 min, 55–70% B; 84–88 min, 70–5% B; 88–90 min, 5% B^59^. Chromatographic data were acquired at 520 nm and the quantification was performed using a cyanidin 3-glucoside standard. The identification of anthocyanins was achieved by comparison of chromatographic retention times and mass spectral information obtained with previously published data. PACs were analyzed by normal-phase HPLC with fluorescence detection using an Agilent 1260/1290 infinity system (Santa Clara, CA). The fluorescence was monitored at excitation and emission wavelengths of 230 nm and 321 nm respectively, and the quantification of polymerization degree (DP) from 1 to >10, was performed using an external calibration curve of epicatechin, applying a correction factor according to their respective responses in fluorescence^59^.

### Experimental design of animal study

All experimental procedures were performed according to the guidelines of the animal care committee of Laval University (CPAUL). The protocols were summited and approved by Canadian Council on Animal Care (CCA). Six-weeks old C57BL/6J male mice (*n* = 72; Jackson laboratories, Sacramento, CA USA) were single-housed in a controlled environment (1 mouse per cage; 12/12-h light-dark) with free access to food and drinking water. After two weeks of acclimation with standard-chow diet (2018 Teklad global 18% protein rodent diet, Harlan laboratories), the mice were randomly divided into six groups (n = 12/group) and fed a Chow diet or a HFHS diet (65% lipids, 15% proteins and 20% carbohydrates). The animals on the control diets (HFHS, and Chow) were treated daily by gavage with the vehicle (water). The polyphenols-treated groups were fed with HFHS-diet and supplemented daily by gavage either with the wild blueberry polyphenol extract (WBE, 200 mg/kg, providing 17 mg of polyphenols per day) or its polyphenolic fractions (BPF), that were rich in anthocyanins and phenolic acids (F1), in oligomeric PACs, phenolic acids and flavonols (F2), or in PACs polymers (F3). In order to determine the contribution of the different blueberry polyphenol classes on the modulation of the gut microbiota composition and the host intestinal and metabolic profile, the amount of BPF administrated to the mice represented the respective proportion of polyphenol of anthocyanins (F1), PACs oligomers (F2), and PACs polymers (F3) contained in the whole WBE. According to the group, the animals received 32 mg/kg of F1 fraction, 53 mg/kg of the F2 fraction, and 37 mg/kg of the F3 fraction (Fig. 1). Body weight gain was taken under non-fasting condition, twice a week. Food intake was evaluated by monitoring the food consumption (g) three times per week. After 5 weeks and 7 weeks of treatment, insulin tolerance test (ITT) and oral glucose tolerance test (OGTT) were performed, respectively. Feces were collected before the start of the treatments (week 0) and at the end (week 8), immediately immersed in dry-ice and stored at −80 °C for subsequent metagenomics analysis. After 8 weeks of feeding, mice were anesthetized with isoflurane (2–3%; 0.5–1.5 L/min) and euthanized by cardiac puncture. Adipose tissues, organs, and intestines were carefully dissected; their weight was recorded and immediately immersed in liquid nitrogen, RNA later (Invitrogen) or into fixation solutions (Carnoy’s solution, buffered formalin 10%) according to the subsequent analysis. Frozen tissues were then stored at −80 °C.

### Glucose homeostasis

After 5 weeks of treatments, mice were fasted for 6 hours. Blood was drawn at baseline and immediately centrifuged (3,500 rpm, 10 min at 4 °C). Insulin solution was administrated (10 μl/g of 0.65U/kg) by intraperitoneal injection, followed by glycemia measurements using an Accu-Check glucometer (Bayer) before and at 5, 10, 15, 20, 30, and 60 minutes after the injection. An oral glucose tolerance test (OGTT) was performed after 7 weeks of dietary treatment. Mice were fasted for 12 hours overnight, blood baseline sample was drawn and immediately centrifuged (3,500 rpm, 10 min at 4 °C). Dextrose solution (2 μl/g of 50% dextrose) was administrated by gavage and blood samples (60 μl) were obtained from the mouse lateral saphenous vein. Glycemia was determined using an Accu-Check glucometer (Bayer) at time 15, 30, 60, 90 and 120 minutes relative to the glucose load. The area under the curve (AUC-OGTT) was determined considering the glucose levels measured between baseline and 120 minutes after glucose overload (GraphPad Prism 7.0, USA). Insulin resistance index (HOMA-IR) was calculated using the formula: HOMA-IR = [fasting glycemia (nmol/L) * fasting insulinemia (µU/mL)/22.5]. The 12-h fasting blood samples were collected for determinations of plasma insulin. Samples were stored at −80 °C until the assay. Mouse insulin was determined using an ultrasensitive ELISA kit (Alpco, Salem, USA).

### Colon histology

Colon tissues were prepared as previously described^60^. The PBS-flushed colon tissues were cut into two equal sized sections (transverse incision). Each section (approximately 0.5 cm) of colon was placed side by side on a piece of tape (3M Micropore surgical tape, 2.5 cm width), and then tightened with forceps. The bundles enclosing the colon pieces were transferred into a tube containing a large excess of cold Methanol-Carnoy’s solution (60% methanol, 30% chloroform and 10% glacial acetic acid), at least 10X the volume of fixative to the volume of tissue. The samples were stored for 3 hr at 4 °C to allow the fixation. After fixation, the tissues were washed with cold 70% ethanol once, and stored in the same solution at 4 °C until they were processed. Tissue processing was performed at IBIS laboratory of Molecular Imaging and Microscopy, Laval University (Québec, QC, Canada). The samples were placed into a tissue cassette indelibly labeled with a unique animal identifier and the name of the intestinal segment. The cassettes were subjected to a tissue processor for standard processing to allow embedding in paraffin wax (Tissue-Tek VIP, vacuum Infiltration Processor Sakura brand). The protocol applied was: Alcohol 95% 45 min, Alcohol 100% 3 × 45 min, Toluene 2 × 45 min, Paraffin 1h30, 2h00 and 4h00. Paraffin-embedded sections of colon tissues were cut and stained with both periodic acid Schiff and Alcian blue (AB-PAS). AB-PAS staining enables the differentiation between acid mucins and neutral mucins, with blue color showing acid mucins, purple color indicating a mixture of neutral and acid mucins, and red/magenta color indicating neutral mucins alone^61^.

### Goblet cell quantification, mucus thickness and crypts depth measurements

The relative proportions and distribution of mucous secreting GC was quantified following Johnson *et al*.^62^ protocols with some modifications^63^. All images were captured using a BX51 microscope (Olympus America, Inc, USA) equipped with a CCD Digital Camera System and Image-Pro software. Number of GC was counted for a defined distance (using 20X objective lens, 50 μm scale). A total of 12 crypts (3 crypts per section) were analyzed for each mouse. Initially, images were taken with the 4X objective lens (200 μm scale bars) in order to examine the whole tissue section. This was used to ensure that the total area of the images taken at a higher magnification would cover at least 50% of the tissue section. Afterwards, under 20X objective lens, each section was divided into four equal quadrants and taking representative images. In each quadrant, within a delineated area (from epithelium towards the colonic crypts), the total GC number was counted, and the data were expressed as the relative cell numbers per crypt (12 crypts of 12 mice per group). To determine the specific mucin cell types for the colon for each animal, the total CG counts were totaled separately (neutral and mixed mucin-filled GC, including mucin unfilled GC) and the data were normalized to reflect the proportion of each mucin cell type per crypt. Crypt lengths (µm) were calculated from an average of 12 crypts per tissue section, for 12 mice. Average of mucus thickness was calculated in a similar manner, measuring the lengths of the inner mucus layer closely adhered to the epithelium, for 12 mice. The National Institute of Health image J software was used for supporting the cell quantifications and crypts length measurements.

### Colon mRNA expression by quantitative RT-PCR

Colon tissues stored in RNA*later* (Invitrogen) at −80 °C were thawed on ice, and total RNA was extracted using a RNeasy mini kit (Qiagen, #74104), according to the manufacturer’s instructions. Samples were DNase treated using the RNase-Free DNase kit (Qiagen; #79254) and RNA concentration and quality was assessed using RNA NanoChips in Agilent 2100 Bioanalyzer (Agilent Technologies, Germany). First-strand cDNA syntheses were prepared from 700 ng of RNA using the RT2 First Strand Kit (Qiagen/SABiosciences). The reaction included one additional step to eliminate traces of genomic DNA. The resultant cDNA product was immediately amplified by qPCR using RT2 SYBR Green qPCR Master Mix (Qiagen/SABiosciences) on an Applied Biosystems ABI 7500 Fast real-time cycling platform. Quantitative RT-PCR was performed in a 25 μl reaction containing 1 μl cDNA, 1 μl of 10 μM reverse and forward primers and 12.5 μl RT2-SYBR Green Master Mix. The thermocycling protocol was as follows: 95 °C for 10 min for hot-start polymerase activation, followed by 40 cycles of denaturation at 95 °C for 15S, annealing at 60 °C for 1 min, and a melting curve stage as default setting from 60 °C to 95 °C. Each sample was analyzed in duplicate and 10–12 biological replicates were utilized for each treatment. For the analysis of the intestinal permeability, ready-made individual primers encoding regulatory genes of differentiation were used (RT2 qPCR Primer Assay, Qiagen). Occludin (*Ocln*, Qiagen #PPM05314A), and Tight junction protein 1 (*Tjp1*, Qiagen #PPM25091A) mouse genes were the targets; Glyceraldehyde-3-phosphate dehydrogenase (*GAPDH*, Qiagen #PPM02946E), Hypoxanthine guanine phosphoribosyl transferase (*Hprt*, Qiagen #PPM03559F), and Peptidylprolyl isomerase B (*Ppib*, Qiagen #PPM03728A) were amplified as housekeeping controls genes (HKGs). Stability of HKGs was further evaluated by geNorm and NormFinder applications. Mouse XpressRef Universal total mRNA (Qiagen #338114) was also used as reference template in order to verify and compare the quality of gene expressions on our mouse experimental mRNA. Data were analyzed using the ΔΔCT method to determine the expression level of each gene normalized to the expression level of the housekeeping gene *Gapdh*.

### Fecal DNA extraction and 16S rRNA gene amplicon library preparation and sequencing

Total DNA was extracted from approximately 100 mg of feces using a ZR fecal DNA kit (D6010; Zymo Research Corp., Orange, CA) which included a bead-beating step for the mechanical lysis of the microbial cells and silica column purification. The DNA extraction was carried out as recommended in the respective kit manuals, with some modifications including a double step of pre-wash and washes during subsequent filtration to remove humic acids/polyphenols that could inhibit PCR. The concentration and quality of DNA was determined spectrophotometrically by measuring the A260/280 using a ND-1000 Nanodrop (Nanodrop Technologies, Wilmington, DE, USA). Fecal DNA sample’s sequencing (12 samples per group diet) were processed at IBIS laboratory, Laval University, Québec, QC, Canada.

Amplicons of the 16S rRNA V3-V4 region were generated using degenerate primers 341F (5′-CCTACGGGNGGCWGCAG-3′) and 805R (5′-GACTACHVGGGTATCTAATCC-3′) adapted to incorporate the transposon-based Illumina Nextera adapters (Illumina, USA) and a sample barcode sequence allowing multiplexed paired-end sequencing. Constructed 16S metagenomic libraries were purified using 35 µL of magnetic beads (AxyPrep Mag PCR Clean up kit; Axygen Biosciences, USA) per 50 µL PCR reaction. Library quality control was performed with a Bioanalyzer 2100 using DNA 7500 chips (Agilent Technologies, USA). An equimolar pool was obtained and checked for quality prior to further processing. The pool was quantified using picogreen (Life Technologies, USA) and loaded on a MiSeq platform using 2 × 300 bp paired-end sequencing (Illumina, USA). High-throughput sequencing was performed at the IBIS (Institut de Biologie Intégrative et des Systèmes - Université Laval).

### 16S rRNA sequence processing

Demultiplexed reads were analyzed using the QIIME software package (version 1.9.1) and customs scripts. The Paired-End read mergeR (PEAR) v.0.9.5^64^ was used to pair the forward and reverse reads of sequences in each sample with at least a 50-bp overlap. Sequences were discarded if they had ambiguous base (N) or a Phred score ≤25 or were shorter than 450 bp in length. Chimeras were filtered with a reference-based approach using UCHIME version 4.2^65^ and a representative set of chimera-checked sequences^66^. The resulting sequences were clustered into OTUs (Operational Taxonomic Units) at 97% identity threshold using an open-reference methodology performed with USEARCH 61 version 6.1.544^67^. Taxonomy of OTUs was assigned to a representative set of 16S rRNA sequences in the Greengenes database using the RDP-classifier^68^. All OTUs that were observed fewer than 2 times (i.e., *singletons*), or with a number of sequences <0.1% of total number of reads were discarded. The RDP classifier against the RDP database release 11^69^ was used to further classified OTUs that were unassigned against Greengenes at the genus level.

### Gut microbiota diversity and composition analysis

OTUs that had prevalence in at least 20% of the mice in a treatment group and had a variance value higher than 10% in a treatment group were included in the analysis. The QIIME α-rarefaction analysis into a depth of 6957 sequences per sample was performed. Individual samples were rarified based on α-diversity estimates, to ensure even sequencing depth for diversity and relative abundance measurements. The α-diversity of each sample was analyzed using the *Shannon-Weaver* diversity index for microbial community composition combining species richness and abundance per sample. The *Chao1* index was determined for richness and observed Species estimation (number of unique OTUs). Non-parametric Mann-Whitney U-tests as well as Kruskal-Wallis test with Benjamini’s multiple comparison correction were conducted to compare diversity between diet types using Prism 8.0 (GraphPad software, California).

β-diversity metrics were calculated using Bray-Curtis distance measure, considering the OTU table rarefied at 6957 sequences per sample for all samples to account for variations in sequencing depth. Principle Coordinate Analyses (PCoA) plots were performed on calculated distance matrices. The statistical significance on β-diversity across sample groups was assessed with the non-parametric Permutational Multivariate Analysis of Variance (PERMANOVA, 999 Monte Carlo permutations) test^70^. PERMANOVA analyses return a *p*-value for significance and also the R^2^-value, which is indicative of the amount of variation attributed to a specific treatment within a model. The significance cutoff for *P* values (PERMANOVA) and corrected *P* values (Kruskal-Wallis) was set at 0.05. Relative frequencies of different taxonomic categories obtained were calculated using the Statistical Analysis of Metagenomic Profiles program (STAMP v.2.1.3).

#### Quantitative real-time PCR analysis

Quantitative real-time Polymerase chain reaction (qPCR) was used to confirm the proportion of specific bacterial taxa found distinctively stimulated on the metagenomic data. *Akkermansia muciniphila* and *Adlercreutzia equolifaciens* relative abundances were analyzed in fecal samples from all groups. Home designed 16S rRNA primer sequences (Geneious software, version 9.0) were used for both *A*. *muciniphila* (forward 5′-CACACCGCCCGTCACAT-3′ and reverse 5′-TGCGGTTGGCTTCAGATACTT-3′), and *A*. *equolifaciens* (forward 5′-TTAGGTAGACGGCGGGGTAA-3′ and reverse 5′-TAGGAGTCTGGGCCGTATCT-3′). Total bacteria were quantified by using the universal primers Uni334F (5′-ACTCCTACGGGAGGCAGCAGT-3′) and Uni514R (5′-ATTACCGCGGCTGCTGGC-3)^71^. Primer amplification efficiencies and standard curves were determined by making dilution series of pure total DNA for each bacterium (ATCC BAA-835 and DSM 19450 strains), calculating a linear regression based on the CT data points, and inferring the efficiency from the slope of the line. Each qPCR was performed in a 20 μl reaction containing 10 μL of 2X PowerUP SYBR Green Master mix (Applied Biosystem), 6.4 μL of water, 0.8 μL of a 5 μM Forward and Reverse primers, and 2 μL of extracted DNA in DNase/RNase-free water. The qPCR amplifications were performed on an Applied Biosystems ABI 7500 Fast real-time cycling platform. The thermocycling protocol consisted in 50 °C for 2 min, 95 °C for 10 min for hot-start polymerase activation, followed by 40 cycles of denaturation at 95 °C for 15 s, annealing at 60 °C for 1 min, and a melting curve stage as default setting from 60 °C to 95 °C. Twelve samples were analyzed for each group, duplicate qPCR reactions were performed. The average CT value obtained from each primer pair was used to calculate the proportion of bacterial taxa over total bacteria in mouse feces. The data were transformed into a percentage using the ∆∆CT-based following formula as described elsewhere^72^:

$$X=\frac{{(Eff.Univ.)}^{CTuniv}}{{(Eff.Spec.)}^{CTspec}}\times 100$$

where *Eff*.*Univ* is the calculated efficiency of the universal primers and *Eff*.*Spec* refers to the efficiency of the taxon-specific primers. *CT univ* and *CT spec* are the CT mean values registered by the Fast real-time thermocycling platform. “X” represents the percentage of 16S taxon-specific copy number existing in each fecal sample. Finally, spearman correlations analyses were performed for comparing the data from 16S rRNA sequencing versus the 16S rRNA relative proportion obtained by qPCR; *p* values < 0.05 were considered statistically significant.

#### Inferred metagenomic functions by PICRUSt

The predictions of metagenome’s functionality, grouped by diet, was predicted using the PICRUSt 1.0.0 software (http://picrust.github.io)^73^. The resulting BIOM table (a collection of closed-reference OTUs) obtained from the QIIME filtered reads and the matched data against a reference collection (GreenGenes database, May 2013 version; http://greengenes.lbl.gov) with 97% identity, was then categorized by KEGG pathways (i.e., KEGG Orthology groups [KOs] were placed into functional categories). The data were analyzed statistically by using STAMP v 2.1.3. LEfSe analysis was performed to identify the microbial functional pathways that were differentially expressed in the different group of diets.

### Statistical analysis

To compute individual pairwise comparisons of means from parametric data, Student’s t-tests and one-way ANOVA with a Dunnett post hoc test (Graph Pad Prism 8) were performed between WBE and BPF groups compared to HFHS group. The significance between groups at different time points was calculated using a two-way repeated measures ANOVA with Dunnett’s post hoc test (Sigma-plot, USA). Kruskal-Wallis or Mann-Whitney U-tests of median were used to analyze nonparametric data as appropriate, considering the samples size and data distribution. The effects of dietary treatments on the abundance of bacterial taxa were analyzed by White’s non-parametric t-test with Benjamini’s correction for multiple comparisons (STAMP; version 2.1.3). To examine differences on the abundance of individual taxa between two groups, a non-parametric Mann-Whitney U-test was applied. *p* values less than 0.05 were considered statistically significant whereas *p values* between 0.05 and 0.1 were considered as showing a trend. Results were expressed as means ± standard error of the mean (SEM).

## Supplementary information

Supplementary information

## Data Availability

The datasets generated during and/or analysed during the current study are available from the corresponding author on reasonable request.
