# Supplementary material for: Wild blueberry proanthocyanidins shape distinct gut microbiota profile and influence glucose homeostasis and intestinal phenotypes in high-fat high-sucrose fed mice
Source: Sci Rep. 2020 Feb 10;10:2217. doi: 10.1038/s41598-020-58863-1 (PMC7010699; doi:10.1038/s41598-020-58863-1)
Supplement: Supplementary file 1 — Supplementary information [file 41598_2020_58863_MOESM1_ESM.pdf]

Supplementary information

Wild blueberry proanthocyanidins shape distinct gut microbiota profile and influence glucose homeostasis and intestinal phenotypes in high-fat high-sucrose fed mice

Maria-Carolina Rodríguez-Daza[1 2 a], Laurence Daoust[1 2 a], Lemia Boutkrabt[1], Geneviève Pilon[1 3], Thibault Varin[1], Stéphanie Dudonné[1], Émile Levy[1], André Marette[1 3], Denis Roy[1 2] and Yves Desjardins[1 2]

[1] Institute of Nutrition and Functional Foods (INAF), Laval University, Québec, QC, Canada.

[2] Food Science Department, Faculty of Agriculture and Food, Laval University, Québec, QC, Canada.

[3] Department of Medicine, Faculty of Medicine, Cardiology Axis of the Quebec Heart and Lung Institute, Québec, QC, Canada.

[a] These authors contributed equally to this work

Corresponding Author:

Yves Desjardins

Institute of Nutrition and Functional Foods (INAF)

Laval University, Québec

Pavillon des Services, # 2736

2440, Boulevard Hochelaga, Québec, QC, Canada G1V 0A6

Telephone: 418-262-0897; email: yves.desjardins@fsaa.ulaval.ca

Table S1. Phenolic characterization of blueberry extract and polyphenolic fractions

	Polyphenols content (mg/100 g dry weight)			
	**Crude extract**	**Polyphenolic fractions**		
**Polyphenol type**	**WBE**	**F1**	**F2**	**F3**
**Anthocyanins**	**840.1 ± 24.8**	**5248.2 ± 427.0**	**29.2 ± 0.8**	**ND**
Delphinidin 3-galactoside	9.6 ± 0.6	57.7 ± 5.5	0.7 ± 0.1	ND
Delphinidin 3-glucoside	28.4 ± 4.6	262.2 ± 47.8	2.2 ± 0.1	ND
Cyanidin 3-galactoside	18.5 ± 2.4	66.5 ± 57.9	1.0 ± 0.1	ND
Delphinidin 3-arabinoside	10.3 ± 2.6	12.7 ± 0.2	0.9 ± 0.2	ND
Cyanidin 3-glucoside	68.8 ± 3.4	612.3 ± 49.3	3.2 ± 0.1	ND
Petunidin 3-galactoside	14.6 ± 1.5	83.1 ± 5.3	0.7 ± 0.3	ND
Cyanidin 3-arabinoside	25.5 ± 2.4	25.8 ± 1.3	1.6 ± 0.03	ND
Petunidin 3-glucoside	53.0 ± 3.3	512.8 ± 40.2	2.4 ± 0.2	ND
Peonidin 3-galactoside	10.3 ± 1.5	48.5 ± 3.4	0.3 ± 0.03	ND
Petunidin 3-arabinoside	14.3 ± 2.3	8.3 ± 0.2	0.7 ± 0.1	ND
Peonidin 3-glucoside	56.5 ± 2.9	554.2 ± 33.8	1.2 ± 0.1	ND
Malvidin 3-galactoside	74.0 ± 5.1	526.7 ± 52.9	1.2 ± 0.2	ND
Malvidin 3-glucoside	207.8 ±4.8	2397.5 ± 198.8	6.8 ± 0.1	ND
Malvidin 3-arabinoside	52.2 ± 4.4	32.3 ± 4.4	ND	ND
Delphinidin 3-(6"-acetoyl) glucoside	11.4 ± 0.5	13.8 ± 1.3	ND	ND
Cyanidin 3-(6"-acetoyl) glucoside	27.0 ± 2.5	7.2 ± 2.1	1.2 ± 0.1	ND
Malvidin 3-(6"-acetoyl) galactoside	26.0 ± 3.1	ND	ND	ND
Petunidin 3-(6"-acetoyl) glucoside	24.0 ± 1.6	ND	1.1 ± 0.2	ND
Peonidin 3-(6"-acetoyl) glucoside	19.2 ± 1.2	7.9 ± 3.2	1.6 ± 0.05	ND
Malvidin 3-(6"-acetoyl) glucoside	88.5 ± 4.1	18.7 ± 3.5	2.2 ± 0.1	ND
**Proanthocyanins**	**3290.4 ± 89.3**	**670.2 ± 83.4**	**7047.7 ± 242.2**	**5520.3 ± 294.2**
Monomers	322.0 ± 8.6	121.4 ± 5.5	1074.4 ± 41.7	27.8 ± 2.2
Dimers	1544.9 ± 24.9	141.1 ± 5.8	4676.1 ± 137.5	89.3 ± 6.5
Trimers	449.3 ± 3.7	27.0 ± 4.1	924.3 ± 35.9	162.5 ± 7.4
Tetramers	255.9 ± 0.5	11.0 ± 1.1	163.9 ± 8.8	412.5 ± 16.1
Pentamers	143.5 ± 3.0	7.3 ± 1.1	ND	296.1 ± 8.7
Hexamers	99.7 ± 4.8	10.4 ± 1.1	ND	210.0 ± 3.7
Heptamers	46.6 ± 3.2	ND	ND	68.2 ± 0.7
Octamers	34.0 ± 1.0	ND	ND	43.2 ± 6.3
Nonamers	26.1 ± 2.5	ND	ND	35.1 ± 5.7
Decamers	16.6 ± 2.5	ND	ND	ND
Polymers >10	351.6 ± 65.1	352.0 ± 64.7	209.0 ± 18.3	4175.5 ± 236.8

**Polyphenols content (mg/100 g dry weight)**				
	**Crude extract**	**Polyphenolic fractions**		
**Polyphenol type**	**WBE**	**F1**	**F2**	**F3**
**Flavonols, flavan-3-nols and phenolic acids**	**4389.5**	**986.06**	**18531.6**	**86.04**
Catechin	97.7 ± 3.6	1.7 ± 1.8	298.2 ± 1.0	5.7 ± 3.4
Epicatechin	75.5 ± 3.1	0.9 ± 0.8	214.1 ± 27.4	8.9 ± 4.3
Gallic acid	115.3 ± 2.2	34.1 ± 2.6	650.2 ± 21.1	1.1 ± 0.2
Protocatechuic acid	355.9 ± 1.6	295.8 ± 9.0	1330.0 ± 80.1	6.6 ± 0.8
P-coumaric acid	116.2 ± 13.3	ND	41.0 ± 7.0	ND
Caffeic acid	134.3 ± 5.3	4.6 ± 0.9	598.7 ± 24.4	ND
Ferulic acid	15.4 ± 2.1	ND	3.2 ± 1.1	ND
3-caffeoylquinic acid	23.8 ± 1.9	0.5 ± 0.5	81.8 ± 11.9	1.4 ± 0.3
4-caffeoylquinic acid	28.4 ± 1.4	0.4 ± 0.2	327.3 ± 8.5	5.3 ± 1.5
5-caffeoylquinic acid	1363.3 ± 42.7	13.0 ± 1.7	4015.3 ± 257.2	27.5 ± 1.5
Quercetin	739.1 ± 17.7	630.9 ± 13.4	4424.1 ± 196.8	11.4 ± 0.8
Quercetin-glucoside	414.5 ± 11.1	0.4 ± 0.4	2273.2 ± 91.0	7.5 ± 1.4
Quercetin-galactoside	135.7 ± 5.3	1.26 ± 0.45	760.7 ± 29.4	2.0 ± 0.6
Quercetin-rhamnoside	250.3 ± 13.0	ND	1422.9 ± 63.3	3.6 ± 2.3
Quercetin-xyloside	133.8 ± 10.6	ND	726.8 ± 64.0	1.1 ± 0.7
Quercetin-arabinoside	101.6 ± 4.2	ND	344.1 ± 8.0	0.4 ± 0.5
Rutin	288.7 ± 3.8	2.5 ± 0.4	1020.0 ± 27.9	3.5 ± 0.6

Results are expressed as mean of triplicate ± SD. ND, not detected. DP, Degree of polymerisation.

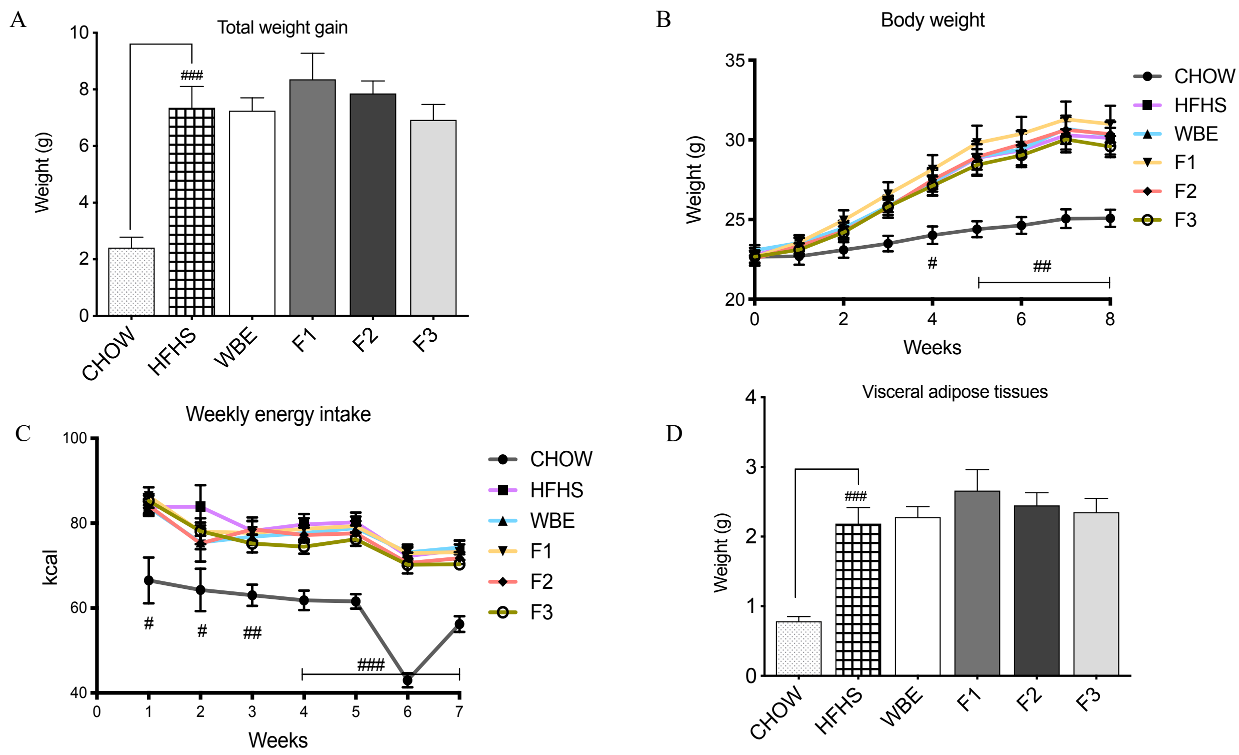

Figure S1. Body weight gain in treated mice are not affected by the administration of a WBE and BPF. C57BL/6J mice were fed an HFHS diet and treated with the vehicle (water), the WBE or a BPF: Fraction rich in anthocyanin and phenolic acids (F1), oligomeric PACs, phenolic acids and flavonols (F2) and polymeric PACs (F3) enriched fractions for 8 weeks. A) Body weight gain; B) weight gain curves; C) energy intake; D) visceral adipose tissue weight. One-way ANOVA with a Dunnett post hoc test was applied to calculate the significance of the differences between groups. Two-way repeated measures RM-ANOVA with a Dunnett post hoc test was applied to calculate the significance between groups at different time points. Values are expressed as the mean ± SEM (n = 12). *p < 0.05 compared to HFHS; Chow vs HFHS # p<0,05 ### p<0,001, #### p<0.0001.

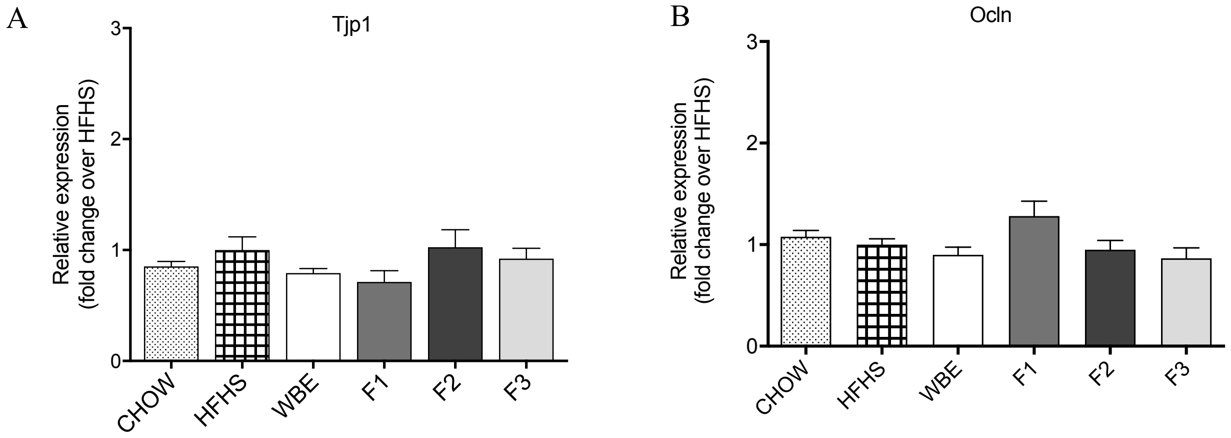

Figure S2. Effects of WBE and BPF on colonic mucin secretion and tight junction integrity of HFHS-fed mice. Relative gene expression determined by RT-qPCR were evaluated in colon tissues of HFHS and BPF fed mice. A) mRNA levels for Tight junction protein 1 (Tjp1) and B) Occludin (Ocln) (n=10-12 per group). Ordinary one-way ANOVA a Dunnett post hoc test was performed, mean +/- SEM. p<0.01## compared to Chow.

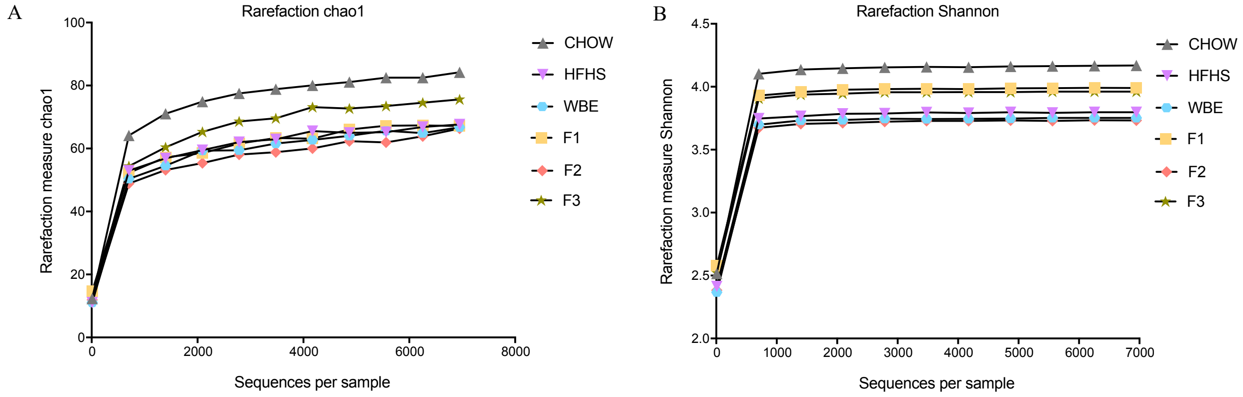

Figure S3. Rarefaction curves graphing within-sample using alpha Chao1 and Shannon diversity measures on mouse fecal bacterial communities. A) Chao1 alpha diversity indicated HFHS-fed mice had reduced species richness than Chow controls. Curves are shown for samples treated with WBE and BPF: anthocyanin and phenolic acids (F1), oligomeric PACs, phenolic acids and flavonols (F2) and polymeric PACs (F3). Kruskal-Wallis test with Benjamini multiple comparison correction were conducted to compare diversity between groups after 8 weeks of dietary treatment. Significant differences were not found between groups (P >0.05 at all sampling depth) B) Shannon diversity considering the abundance and richness showed curves similar depth of sequences among HFHS-fed mice and polyphenols treated mice.

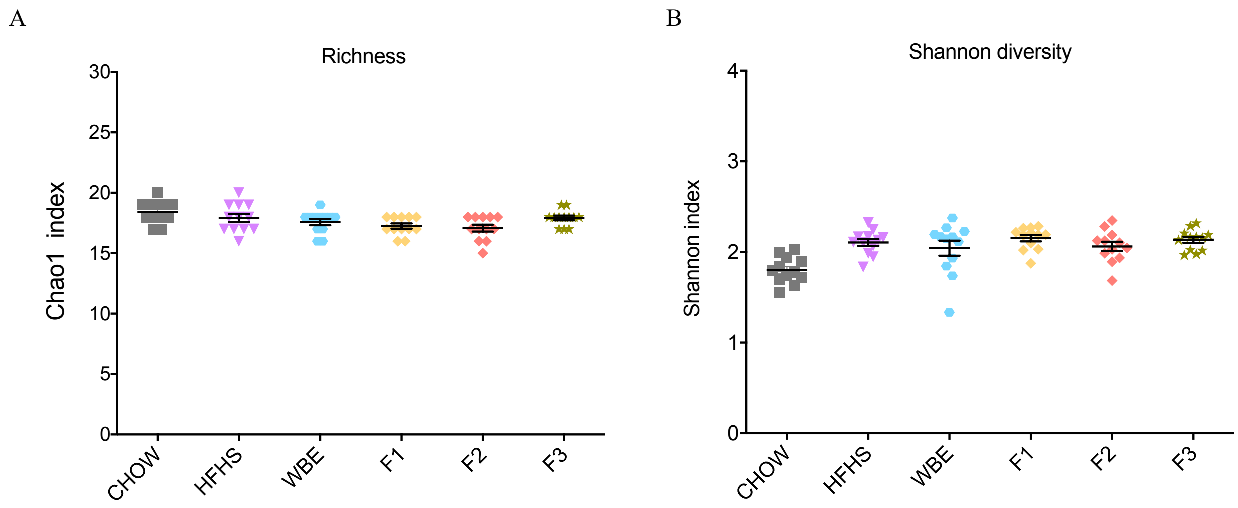

Figure S4. Alpha diversity indices. The effect of HFHS-diet and the supplementation with WBE and BPF: anthocyanin and phenolic acids (F1), oligomeric PACs, phenolic acids and flavonols (F2) and polymeric PACs (F3) on the microbial alpha diversity were studied. A) Richness: the number of bacterial species assigned by OTUs detected in the samples was obtained using Chao1 index. B) Shannon's diversity index: Incorporates both richness and evenness. Kruskal-Wallis test with Benjamini multiple comparison correction were conducted to compare diversity between diets after 8 weeks of feeding. Mean values ±SEM are plotted, no significant differences were found.

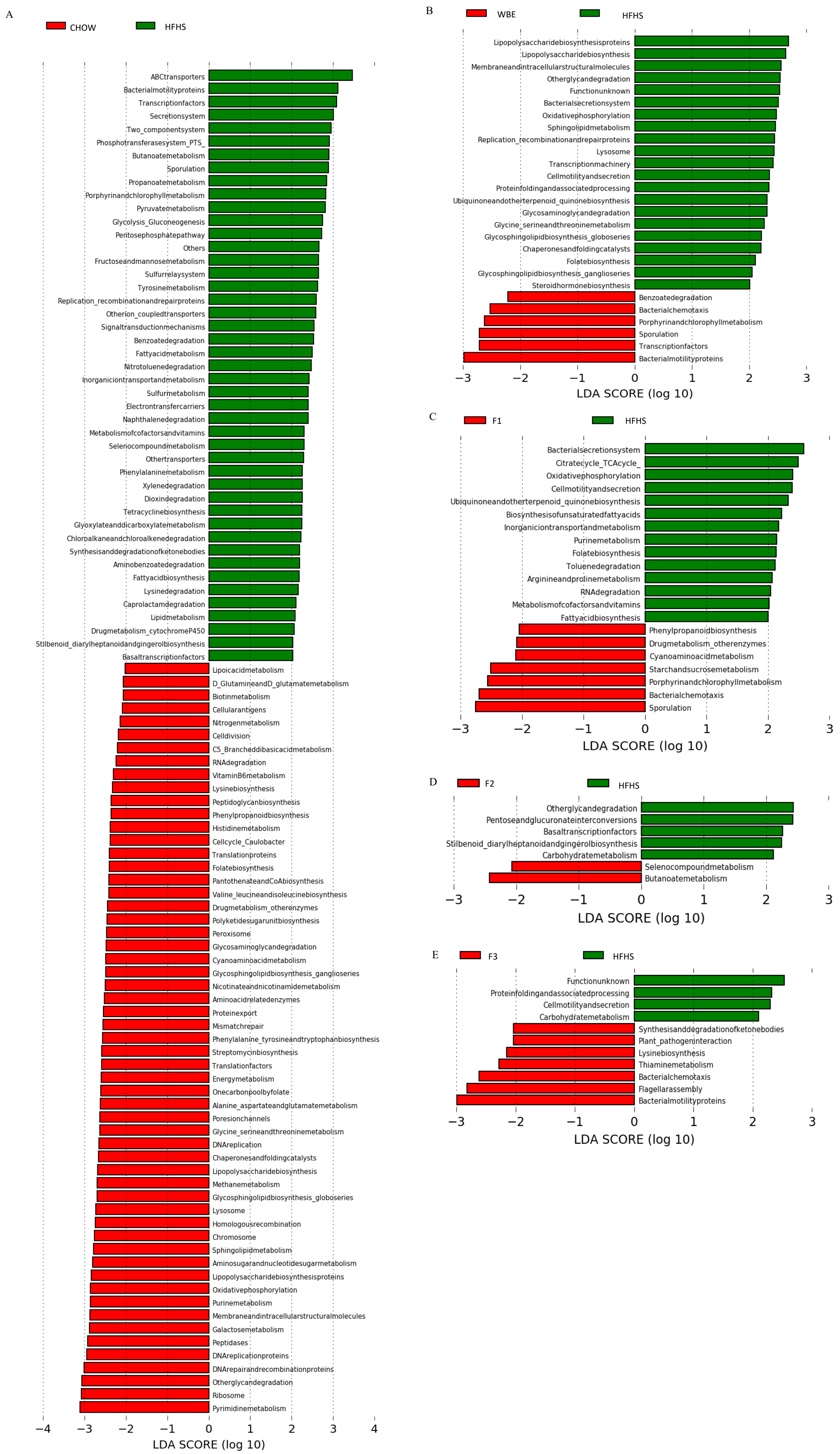
A
CHOW
HFHS
ABCtransporters
Bacterialmotilityproteins
Transcriptionfactors
Secretionsystem
Two_componentsystem
Phosphotransferasesystem_PTS_
Butanoatemetabolism
Sporulation
Propanoatemetabolism
Porphyrinandchlorophyllmetabolism
Pyruvatemetabolism
Glycolysis_Gluconeogenesis
Pentosephosphatepathway
Others
Fructoseandmannosemetabolism
Sulfurrelaysystem
Tyrosinemetabolism
Replication_recombinationandrepairproteins
Otherion_coupledtransporters
Signaltransductionmechanisms
Benzoatedegradation
Fattyacidmetabolism
Nitrotoluenedegradation
Inorganiciontransportandmetabolism
Sulfurmetabolism
Electrontransfercarriers
Naphthalenedegradation
Metabolismofcofactorsandvitamins
Selenocompoundmetabolism
Othertransporters
Phenylalaninemetabolism
Xylenedegradation
Dioxindegradation
Tetracyclinebiosynthesis
Glyoxylateanddicarboxylatemetabolism
Chloroalkaneandchloroalkenedegradation
Synthesisanddegradationofketonebodies
Aminobenzoatedegradation
Fattyacidbiosynthesis
Lysinedegradation
Caprolactamdegradation
Lipidmetabolism
Drugmetabolism_cytochromeP450
Stilbenoid_diarylheptanoidandgingerolbiosynthesis
Basaltranscriptionfactors
Lipoicacidmetabolism
D_GlutamineandD_glutamatemetabolism
Biotinmetabolism
Cellularantigens
Nitrogenmetabolism
Celldivision
C5_Branchedbasicacidmetabolism
RNAdegradation
VitaminB6metabolism
Lysinebiosynthesis
Peptidoglycanbiosynthesis
Phenylpropanoidbiosynthesis
Histidinemetabolism
Cellcycle_Caulobacter
Translationproteins
Folatebiosynthesis
PantothenateandCoAbiosynthesis
Valine_leucineandisoleucinebiosynthesis
Drugmetabolism_otherenzymes
Polyketidesugarunitbiosynthesis
Peroxisome
Glycosaminoglycandegradation
Cyanoaminoacidmetabolism
Glycosphingolipidbiosynthesis_ganglioseries
Nicotinateandnicotinamidemetabolism
Aminoacidrelatedenzymes
Proteinexport
Mismatchrepair
Phenylalanine_tyrosineandtryptophanbiosynthesis
Streptomycinbiosynthesis
Translationfactors
Energymetabolism
Onecarbonpoolbyfolate
Alanine_aspartateandglutamatemetabolism
Poresionchannels
Glycine_serineandthreoninemetabolism
DNAreplication
Chaperonesandfoldingcatalysts
Lipopolysaccharidebiosynthesis
Methanemetabolism
Glycosphingolipidbiosynthesis_globoseries
Lysosome
Homologousrecombination
Chromosome
Sphingolipidmetabolism
Aminosugarandnucleotidesugarmetabolism
Lipopolysaccharidebiosynthesisproteins
Oxidativephosphorylation
Purinemetabolism
Membraneandintracellularstructuralmolecules
Galactosemetabolism
Peptidases
DNAreplicationproteins
DNArepairandrecombinationproteins
Otherglycandegradation
Ribosome
Pyrimidinemetabolism
−4 −3 −2 −1 0 1 2 3 4
LDA SCORE (log 10)
B
WBE
HFHS
Lipopolysaccharidebiosynthesisproteins
Lipopolysaccharidebiosynthesis
Membraneandintracellularstructuralmolecules
Otherglycandegradation
Functionunknown
Bacterialsecretionsystem
Oxidativephosphorylation
Sphingolipidmetabolism
Replication_recombinationandrepairproteins
Lysosome
Transcriptionmachinery
Cellmotilityandsecretion
Proteinfoldingandassociatedprocessing
Ubiquinoneandotherterpenoid_quinonebiosynthesis
Glycosaminoglycandegradation
Glycine_serineandthreoninemetabolism
Glycosphingolipidbiosynthesis_globoseries
Chaperonesandfoldingcatalysts
Folatebiosynthesis
Glycosphingolipidbiosynthesis_ganglioseries
Steroidhormonebiosynthesis
Benzoatedegradation
Bacterialchemotaxis
Porphyrinandchlorophyllmetabolism
Sporulation
Transcriptionfactors
Bacterialmotilityproteins
−3 −2 −1 0 1 2 3
LDA SCORE (log 10)
C
F1
HFHS
Bacterialsecretionsystem
Citratecycle_TCAcycle_
Oxidativephosphorylation
Cellmotilityandsecretion
Ubiquinoneandotherterpenoid_quinonebiosynthesis
Biosynthesisofunsaturatedfattyacids
Inorganiciontransportandmetabolism
Purinemetabolism
Folatebiosynthesis
Toluenedegradation
Arginineandprolinemetabolism
RNAdegradation
Metabolismofcofactorsandvitamins
Fattyacidbiosynthesis
Phenylpropanoidbiosynthesis
Drugmetabolism_otherenzymes
Cyanoaminoacidmetabolism
Starchandsucrosemetabolism
Porphyrinandchlorophyllmetabolism
Bacterialchemotaxis
Sporulation
−3 −2 −1 0 1 2 3
LDA SCORE (log 10)
D
F2
HFHS
Otherglycandegradation
Pentoseandglucuronateinterconversions
Basaltranscriptionfactors
Stilbenoid_diarylheptanoidandgingerolbiosynthesis
Carbohydratemetabolism
Selenocompoundmetabolism
Butanoatemetabolism
−3 −2 −1 0 1 2 3
LDA SCORE (log 10)
E
F3
HFHS
Functionunknown
Proteinfoldingandassociatedprocessing
Cellmotilityandsecretion
Carbohydratemetabolism
Synthesisanddegradationofketonebodies
Plant_pathogeninteraction
Lysinebiosynthesis
Thiaminemetabolism
Bacterialchemotaxis
Flagellarassembly
Bacterialmotilityproteins
−3 −2 −1 0 1 2 3
LDA SCORE (log 10)

Figure S5. LEfse analysis of fecal microbial functional profiles between HFHS-fed mice and wild blueberry polyphenols treated mice. LDA scores for the bacterial taxa differentially abundant between HFHS-fed mice, WBE and BPF: anthocyanin and phenolic acids (F1), oligomeric PACs, phenolic acids flavonols (F2) and polymeric PACs (F3). Positive and negative LDA scores indicate the bacterial taxa enriched in A) WBE-fed mice vs HFHS, B) F1-fed mice vs HFHS, C) F2 vs HFHS and D) F3 vs HFHS. Only the taxa having a $p < 0.05$ (Wilcoxon-Mann Whitney rank-sum test) and LDA >2.0 are shown in the figure legend.
